# One-pot synthesis of chromenes in the presence of nano-cellulose/Ti^(IV)^/Fe_3_O_4_ as natural-based magnetic nano-catalysts under solvent free conditions†

**DOI:** 10.1039/d2ra05057a

**Published:** 2022-09-28

**Authors:** Raziyeh Gholami, Abdolhamid Bamoniri, Bi Bi Fatemeh Mirjalili

**Affiliations:** Department of Organic Chemistry, Faculty of Chemistry, University of Kashan Kashan I. R. Iran bamoniri@kashanu.ac.ir +98-31-55912384; Department of Chemistry, Faculty of Science, Yazd University Yazd I. R. Iran

## Abstract

In this study, the preparation of magnetic catalysts of titanium tetrachloride stabilized on nano-cellulose named cellulose/Ti^(IV)^/Fe_3_O_4_ was investigated. Various methods such as XRD, SEM, FT-IR, BET, EDX, TEM, TGA and VSM were used to characterize the catalysts. Then, the identified catalysts were used for the synthesis of various chromene skeletons *via* reaction of malononitrile, aldehyde and dimedone, 4-hydroxycoumarine or 2-naphthole at 70 °C under solvent free conditions. The spectroscopic methods used to determine the structure of the products include ^13^C NMR, ^1^H NMR and FT-IR.

## Abstract 2

In this study, the preparation of magnetic catalysts of titanium tetrachloride stabilized on nano-cellulose named cellulose/Ti^(IV)^/Fe_3_O_4_ was investigated.

## Graphical abstract

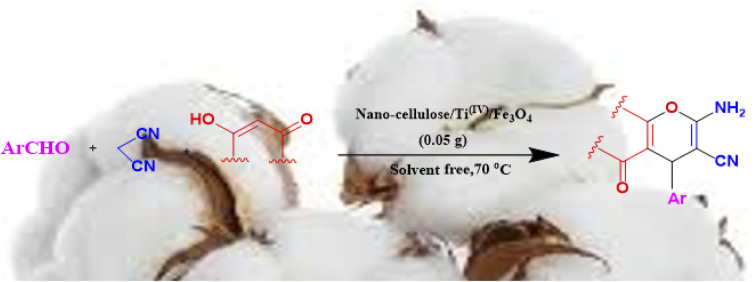

## Introduction

Multi-component reactions (MCRs) are important and useful tools for producing complex molecules from simple raw materials. In these reactions, three or more simple raw materials participate in a condensation reaction to form more complex organic molecules through the formation of carbon–carbon or carbon–heteroatom bonds. MCRs are performed simultaneously and in a one-pot reaction, and without the separation of intermediates the reaction efficiency increases significantly. This type of reaction saves time, energy and raw materials. They also have other advantages such as making new multiple bonds, high efficiency, saving on solvents and chemicals, reducing by-product production, simple product separation and modest purification. MCRs are a useful method for the synthesis of a variety of molecules that take place without the separation of intermediates.

Catalysts have become a strategic part of modern science. Nano-dimensions have provided ideal conditions for catalyst science. Active levels and excellent selectivity in nano-catalysts have led to increased reaction speed and efficiency. Among magnetic nano-particles, Fe_3_O_4_ nano-particles have a higher capacity for better recycling and are used as a support in the synthesis of organic compounds. In recent years, the focus has been on magnetic nano-catalysts for the synthesis of heterocyclic compounds and subsequent drug production.

Heterocyclic compounds play an important role in drug synthesis. Among them, the chromenes are very significant. Chromenes are an important class of oxygenated heterocyclic compounds that have gained considerable importance due to their reactivity, diverse biological activity, and therapeutic applications.^1,2^ Among the biological properties of chromenes,^3^ we can mention the antimicrobial^4,5^ and inhibitory properties of influenza virus, antitumor, effect on the nervous system in the treatment of Alzheimer's, hypertension and seizures and anti-HIV properties. Due to the research done and the widespread use of chromene derivatives as pharmaceutical and biologically active compounds and the development of green chemistry to provide an easy, effective and rapid way to prepare this family of compounds is very important. However, various methods have been reported for the preparation of these compounds. But due to problems such as solvent use, long reaction time, toxicity and non-recovery, they have been replaced by new methods. The use of magnetic catalysts in nano-dimensions, in comparison with other catalysts presented for the preparation of these compounds are very suitable and has been considered by many researchers today.^6,7^ Increasing the efficiency and reducing the reaction time by using environmentally friendly catalysts, and also preparing high-purity organic compounds were the main reasons for this research. Herein, we report a simple and efficient method for the synthesis and preparation of various chromenes using various aldehydes by natural based nano-cellulose/Ti^(IV)^/Fe_3_O_4_ catalyst.

## Experimental

### Materials and apparatus

All chemicals were obtained from Merck and Fluka companies and used without any additional purification. FT-IR spectra were obtained on a Magna 550 Nicolet spectrometers. A Bruker (DRX-400 Avance) NMR was used to record the ^1^H NMR spectra. Melting points were determined by a Buchi melting point B-540 B.V.CHI apparatus. XRD pattern was achieved on Philips Xpert MP diffract meter (Cu Kα, radiation, *k* = 0.154056 nm). FESEM was obtained on a Mira Tescan, Phenom pro X. BET surface area analysis was done with micrometrics, Tristar II 3020 analyzer. TGA was done with STA 504 instrument. Sonication was performed in Kunshan KQ-250B ultrasonic reactor with a frequency of 40 kHz and a nominal power of 250 W. The products were characterized by FT-IR, ^1^H NMR, and a comparison of their physical properties with those reported in the literature.

### Preparation of nano-cellulose/Ti^(IV)^/Fe_3_O_4_ nano-particles

Firstly, for preparation of nano-cellulose/Ti^(IV)^, an amount of 0.5 mL of TiCl_4_ was added dropwise to a mixture of nano-cellulose (0.5 g) in 5 mL of dichloromethane and stirred for 1 h at room temperature. Then, the resulting mixture was filtered and washed with dichloromethane and dried at room temperature. Subsequently, the resulting nano-cellulose/Ti^(IV)^ with 0.5 g of Fe_3_O_4_ nano-particles was dispersed in 5 mL of dichloromethane under ultrasound irradiation at room temperature for 1 h. Then, the resulting suspension was filtered and washed with dichloromethane and dried at room temperature so that nano-cellulose/Ti^(IV)^/Fe_3_O_4_ catalyst was obtained.

General procedure for the synthesis of chromens in the presence of nano-cellulose/Ti^(IV)^/Fe_3_O_4_ as nano-catalyst under solvent-free conditions.

In a 100 mL flask, a mixture of aromatic aldehyde (1 mmol), malononitrile (1.5 mmol) and 1,3-diketone (1 mmol) in the presence of 0.012 g of nano-cellulose/Ti^(IV)^/Fe_3_O_4_ was added. The mixture was stirred for the required time at 70 °C. During the reaction, its progression was followed by thin layer chromatography (TLC, ethyl acetate :  hexane, 3 : 7). At the end of the reaction, the mixture was cooled to room temperature and then the catalyst was separated from the reaction solution by an external magnet. For further purification, the product was recrystallized from ethanol, and at the end the product was washed three times with 10 mL of cold diethyl ether. The isolated catalyst was also rinsed several times with chloroform and hot ethanol for reuse.

#### 2-Amino-7,7-dimethyl-5-oxo-4-phenyl-5,6,7,8-tetrahydro-4*H*-chromene-3-carbonitrile (4a)

Milky solid; mp = 238–240 °C; IR (KBr) *ν̄* (cm^−1^): 3395, 3324 (NH_2_), 3083, 3028 (C_Ar–H_), 2198 (C≡N), 1661 (C=O), 1602 (C=C), 1035 (C–O); ^1^H-NMR (DMSO-d_6_, 400 MHz) *δ* (ppm):0.94 (s, 3H, CH_3_), 1.02 (s, 3H, CH_3_), 2.08 (m, 2H, CH_2_), 2.24 (m, 2H, CH_2_), 4.15 (s, 1H, CH), 6.97 (s, 2H, NH_2_), 7.14 (t, *J* = 7.12 Hz, 3H, Ar–H), 7.25–7.28 (m, 2H, Ar–H).

#### 2-Amino-4-(4-hydroxy-3-methoxyphenyl)-7,7-dimethyl-5-oxo-5,6,7,8 tetrahydro-4*H*-chromene-3-carbonitrile (4b)

White solid; mp = 230–231 °C; IR (KBr) *ν̄* (cm^−1^): 3497 (O–H), 3403, 3324 (NH_2_), 3016 (Ar–H), 2192 (C≡N), 1654 (C=O), 1603 (C=C), 1034 (C–O); ^1^H-NMR (DMSO-d_6_, 400 MHz) *δ* (ppm): 0.95 (s, 3H, CH_3_), 1.02 (s, 3H, CH_3_), 2.08 (m, 2H, CH_2_), 2.23 (m, 2H, CH_2_), 3.69 (s, 3H, OCH3), 4.05 (s, 1H, CH), 6.48–6.51 (m, 1H), 6.62–6.66 (m, 2H), 6.89 (s, 2H, NH_2_), 8.80 (s, 1H, OH).

#### 2-Amino-4-(4-chlorophenyl)-7,7-dimethyl-5-oxo-5,6,7,8-tetrahydro-4*H*-chromene-3 carbonitrile (4c)

White crystal; mp = 210–212 °C; IR (KBr) *ν̄* (cm^−1^): 3380, 3323 (NH_2_), 3183, 2959 (Ar–H), 2188 (C≡N), 1676 (C=O), 1603 (C=C), 1032 (C–O); ^1^H-NMR (DMSO-d_6_, 400 MHz) *δ* (ppm): 0.92 (s, 3H, CH_3_), 1.01 (s, 3H, CH_3_), 2.08 (m, 2H, CH_2_), 2.22 (m, 2H, CH_2_), 4.17 (s, 1H, CH), 7.03 (s, 2H, NH_2_), 7.15 (d, *J* = 8.44 Hz, 2H), 7.32 (d, *J* = 8.4 Hz, 1H).

#### 2-Amino-4-(2,4-dimethoxyphenyl)-7,7-dimethyl-5-oxo-5,6,7,8-tetrahydro-4*H*-chromene-3-carbonitrile (4d)

Yellowish white; mp = 229–231 °C; IR (KBr) *ν̄* (cm^−1^): 3390, 3326 (NH_2_), 3256, 3213, 2954 (Ar–H), 2193 (C≡N), 1657 (C=O), 1604 (C=C), 1031 (C–O); ^1^H-NMR (DMSO-d_6_, 400 MHz) *δ* (ppm):0.96 (3H, s, CH_3_), 1.01 (3H, s, CH_3_), 2.07 (m, 2H, CH_2_), 2.21 (m, 2H, CH_2_), 3.68 (6H, s, OCH_3_), 4.09 (1H, s, CH), 6.83 (4H, br s, H–Ar, NH_2_).

#### 2-Amino-7,7-dimethyl-4-(2-nitrophenyl)-5-oxo-5,6,7,8-tetrahydro-4*H*-chromene-3-carbonitrile (4e)

Milky solid; mp = 236–238 °C; IR (KBr) *ν̄* (cm^−1^):3471, 3332 (NH_2_), 3255, 3210, 2960 (Ar–H), 2194 (C≡N), 1688 (C=O), 1602 (C=C), 1525, 1596 (NO_2_), 1041 (C–O); ^1^H-NMR (DMSO-d_6_, 400 MHz) *δ* (ppm):0.81 (s, 3H, CH_3_), 0.94 (s, 3H, CH_3_), 1.94 (m, 2H, CH_2_), 2.13 (m, 2H, CH_2_), 4.86 (s, 1H), 7.11 (s, 2H, NH_2_), 7.28 (dd, *J* = 6.78, 1.00 Hz, 1H), 7.36 (t, *J* = 7.2 Hz, 1H), 7.57–7.75 (m, 2H).

#### 2-Amino-4-(2-methoxyphenyl)-7,7-dimethyl-5-oxo-5,6,7,8-tetrahydro-4*H*-chromene-3-carbonitrile (4f)

Light brown solid; mp = 195–197 °C; IR (KBr) *ν̄* (cm^−1^):3396, 3329 (NH_2_), 3262, 3219, 2964 (Ar–H), 2189 (C≡N), 1685 (C=O), 1654 (C=C), 1036 (C–O); ^1^H-NMR (DMSO-d_6_, 400 MHz) *δ* (ppm): 0.95 (3H, s, CH_3_), 1.02 (3H, s, CH_3_), 2.14 (m, 2H, CH_2_), 3.73 (3H, s, OCH_3_), 4.45 (1H, s, CH), 6.82 (2H, br s, NH_2_), 6.96 (2H, br s, H–Ar), 7.14 (1H, br s, H–Ar).

#### 4,4′-(1,4-Phenylene)bis(2-amino-7,7-dimethyl-5-oxo-5,6,7,8-tetrahydro-4*H*-chromene-3 carbonitrile) (4g)

White solid; mp = >280 °C; IR (KBr) *ν̄* (cm^−1^):3392, 3325 (NH_2_), 3254, 3211, 2963 (Ar–H), 2194 (C≡N), 1686 (C=O), 1649 (C=C), 1041 (C–O); ^1^H-NMR (DMSO-d_6_, 400 MHz) *δ* (ppm): 0.93 (3H, s, CH_3_), 1.02 (3H, s, CH_3_), 2.15 (m, 2H, CH_2_), 4.24 (1H, s, CH), 7.08 (2H, br s, NH_2_), 7.25 (2H, d, *J* = 8, H–Ar), 7.85 (2H, d, *J* = 8, H–Ar).

#### 2-Amino-5-oxo-4-(*m*-tolyl)-4,5-dihydropyrano[3,2-*c*]chromene-3-carbonitrile (4h)

White solid; mp = 253–255 °C; IR (KBr) *ν̄* (cm^−1^):3390, 3321 (NH_2_), 3050, 3015 (Ar–H), 2198 (C≡N), 1705, 1674 (C=O), 1603 (C=C), 1060 (C–O); ^1^H-NMR (DMSO-d_6_, 400 MHz) *δ* (ppm): 2.24 (3H, s, CH_3_), 4.42 (1H, s, CH), 7.03 (3H, br s, NH_2_), 7.183 (1H, t, *J* = 8, H–Ar), 7.379 (1H, s, H–Ar), 7.445 (1H, d, *J* = 11, H–Ar), 7.49 (1H, d,*J* = 9.5, H–Ar), 7.69 (1H, t, *J* = 9.5, H–Ar), 7.89 (1H, d, *J* = 9.5,H–Ar).

#### 2-Amino-5-oxo-4-(2-chlorophenyl)-4,5-dihydropyrano[3,2-*c*] chromene-3 carbonitrile (4i)

Milky solid; mp = 265–267 °C; IR (KBr) *ν̄* (cm^−1^): 3398, 3284 (NH_2_), 3179 (Ar–H), 2199 (C≡N), 1708, 1673 (C=O), 1603 (C=C), 1060 (C–O); ^1^H-NMR (DMSO-d_6_, 400 MHz) *δ* (ppm):4.96 (1H, s, CH), 7.28 (3H, m, NH_2_, H–Ar), 7.47 (4H, m, H–Ar), 7.71 (1H, t, *J* = 9.5, H–Ar), 7.89 (1H, d, *J* = 9.5, H–Ar).

#### 2-Amino-5-oxo-4-phenyl-4,5-dihydropyrano[3,2-*c*]chromene-3-carbonitrile (4j)

Light brown solid; mp = 259–261 °C; IR (KBr) *ν̄* (cm^−1^): 3377, 3285 (NH_2_), 3180, 2887 (Ar–H), 2196 (C≡N), 1709, 1673 (C=O), 1605 (C=C), 1056 (C–O); ^1^H-NMR (DMSO-d_6_, 400 MHz) *δ* (ppm):4.43 (1H, s, CH), 7.24–7.47 (9H, m, NH_2,_ H–Ar), 7.69 (1H, br s, H–Ar), 7.88 (1H, d, *J* = 6.5, H–Ar).

#### 2-Amino-5-oxo-4-(4-nitrophenyl)-4,5-dihydropyrano[3,2-*c*] chromene-3-carbonitrile (4k)

White solid; mp = 252–254 °C; IR (KBr) *ν̄* (cm^−1^): 3335–3479 (NH_2_), 3191, 3069 (Ar–H), 2195 (C≡N), 1718, 1672 (C=O), 1602 (C=C), 1505, 1456 (NO_2_), 1054 (C–O); ^1^H-NMR (DMSO-d_6_, 400 MHz) *δ* (ppm):4.66 (1H, s, CH), 7.45–7.59 (4H, m, NH_2,_ H–Ar), 7.72 (1H, t, *J* = 10, H–Ar), 7.90 (1H, d, *J* = 10, H–Ar), 8.16 (1H, d, *J* = 10.5, H–Ar).

#### 2-Amino-4-(4-methoxyphenyl)-5-oxo-4,5-dihydropyrano[3,2-*c*] chromene-3-carbonitrile (4l)

Yellow solid; mp = 246–248 °C; IR (KBr) *ν̄* (cm^−1^): 3383, 3319 (NH_2_), 3252, 3190, 3064, 2954 (Ar–H), 2202 (C≡N), 1709, 1672 (C=O), 1606 (C=C), 1052 (C–O); ^1^H-NMR (DMSO-d_6_, 400 MHz) *δ* (ppm):3.70 (3H, s, OCH_3_), 4.38 (1H, s, CH), 6.85 (2H, d, *J* = 11,_,_ H–Ar), 7.15 (2H, d, *J* = 11, H–Ar), 7.36 (2H, br s, NH_2_), 7.43 (1H, d, *J* = 10.5, H–Ar), 7.48 (1H, d, *J* = 9.5, H–Ar), 7.69 (1H, t, *J* = 9.5, H–Ar), 7.88 (1H, d, *J* = 10, H–Ar).

#### 4,4′-(1,4-Phenylene)bis(2-amino-5-oxo-4,5-dihydropyrano[3,2-*c*] chromene-3-carbonitrile) (4m)

Light yellow solid; mp = >280 °C; IR (KBr) *ν̄* (cm^−1^): 3324 (NH_2_), 3191 (Ar–H), 2196 (C≡N), 1709, 1672 (C=O), 1604 (C=C), 1055 (C–O);^1^H-NMR (DMSO-d_6_, 400 MHz) *δ* (ppm):4.40 (1H, s, CH), 7.26 (4H, br s, NH_2_), 7.31 (3H, d, *J* = 6.0 Hz, H–Ar), 7.42 (3H, br s, H–Ar), 7.47 (2H, d, *J* = 10.8 Hz, H–Ar), 7.90 (2H, d, *J* = 6.8 Hz, H–Ar), 7.70 (2H, br s, H–Ar).

#### 2-Amino-4-(4-hydroxy-3-methoxyphenyl)-7-methyl-5-oxo-4,5dihydropyrano[4,3-*b*]pyran-3-carbonitrile(4n)

White solid; mp = 260–262 °C; IR (KBr) *ν̄* (cm^−1^): 3492 (OH), 3349, 3094 (NH_2_), 3094 (Ar–H), 2200 (C≡N), 1702, 1673 (C=O), 1639, 1607 (C=C), 1034 (C–O); ^1^H-NMR (DMSO-d_6_, 400 MHz) *δ* (ppm): 2.22 (3H, s, CH_3_), 3.74 (3H, s, OCH_3_), 4.16 (1H, s, CH), 6.24 (1H, s, H-pyran), 6.51 (1H, d, *J* = 9, H–Ar), 6.68 (1H, d, *J* = 10.5, H–Ar), 6.72 (1H, s, H–Ar), 7.12 (2H, br s, NH_2_), 8.92 (1H, br s, OH).

#### 2-Amino-7-methyl-4-(3-nitrophenyl)-5-oxo-4,5-dihydropyrano[4,3-*b*]pyran-3-carbonitrile (4o)

White solid; mp = 236–238 °C; IR (KBr) *ν̄* (cm^−1^):3457, 3360 (NH_2_), 3237, 3184, 3118, 3076 (Ar–H), 2874, 2195 (C≡N), 1705, 1670 (C=O), 1639, 1609 (C=C), 1473, 1526 (NO_2_), 1038 (C–O); ^1^H-NMR (DMSO-d_6_, 400 MHz) *δ* (ppm): 2.21 (3H, s, CH_3_), 4.54 (1H, s, CH), 6.30 (1H, s, H-pyran), 7.35 (1H, s, H–Ar), 8.03 (2H, s, NH_2_), 8.11 (1H, d, *J* = 10, H–Ar).

#### 2-Amino-4-(4-ethoxyphenyl)-7-methyl-5-oxo-4,5-dihydropyrano[4,3-*b*]pyran-3-carbonitrile (4p)

White solid; mp = 230–232 °C; IR (KBr) *ν̄* (cm^−1^): 3450, 3401 (NH_2_), 3209 (C–H), 3101, 2980 (Ar–H), 2929, 2883, 2195 (C≡N), 1702, 1671 (C=O), 1643, 1610 (C=C), 1042 (C–O); ^1^H-NMR (DMSO-d_6_, 400 MHz) *δ* (ppm): 1.28 (3H, t, *J* = 8, CH_3_), 2.19 (3H, s, CH_3_), 3.96 (2H, q, *J* = 7.5, CH_2_), 4.19 (1H, s, CH), 6.24 (1H, s, H-pyran), 6.82 (2H, d, *J* = 10, H–Ar), 7.06 (2H, d, *J* = 10, H–Ar), 7.14 (2H, br s, NH_2_).

#### 4,4′-(1,4-Phenylene)bis(2-amino-7-methyl-5-oxo-4,5-dihydropyrano [4,3-*b*]pyran-3-carbonitrile) (4q)

Yellowish white solid; mp = >280 °C; IR (KBr) *ν̄* (cm^−1^): 3457, 3323 (NH_2_), 3196 (Ar–H), 2891 (aliphatic C–H), 2195 (C≡N), 1704, 1677 (C=O), 1643, 1610 (C=C), 1039 (C–O); ^1^H-NMR (DMSO-d_6_, 400 MHz) *δ* (ppm): 2.19 (3H, s, CH_3_), 4.22 (1H, s, CH), 6.25 (1H, s, H-pyran), 7.09 (2H, s, NH_2_), 7.18 (4H, s, H–Ar).

#### 3-Amino-1-(3-nitrophenyl)-1*H*-benzo[*f*]chromene-2-carbonitrile (4r)

Milky solid; mp = 238–240 °C; IR (KBr) *ν̄* (cm^−1^): 3404, 3356 (NH_2_), 3196, 2891 (Ar–H), 2190 (C≡N), 1658 (C=O), 1643, 1610 (C=C), 1527, 1349 (NO_2_), 1083 (C–O); ^1^H-NMR (DMSO-d_6_, 400 MHz) *δ* (ppm): 5.40 (1H, s, CH), 7.20 (2H, s, NH_2_), 7.42 (2H, d, *J* = 9.01 Hz, Ar–H), 7.51–7.57(2H, m, Ar–H), 7.63 (1H, d, *J* = 7.74 Hz, Ar–H), 7.78 (1H, d, *J* = 9.11 Hz, Ar–H), 7.93 (1H, d, *J* = 9.06 Hz, Ar–H), 8.01 (1H, d, *J* = 8.01 Hz, Ar–H), 8.06 (1H, s, Ar–H), 8.05 (1H, d, *J* = 1.75 Hz, Ar–H).

#### 3-Amino-1-(*p*-tolyl)-1*H*-benzo[*f*]chromene-2-carbonitrile (4s)

White solid; mp = 268–270 °C; IR (KBr) *ν̄* (cm^−1^): 3404, 3356 (NH_2_), 3079 (Ar–H), 2190 (C≡N), 1658 (C=O), 1615 (C=C), 1035 (C–O); ^1^H-NMR (DMSO-d_6_, 400 MHz) *δ* (ppm): 2.17 (3H, s, CH_3_), 5.22 (1H, s, CH), 6.25 (1H, s, CH), 6.94 (1H, br s, H–Ar), 7.04 (4H, br s, H–Ar), 7.30–7.40 (8H, m, H–Ar, NH_2_), 7.80–7.89 (3H, m, H–Ar).

## Results and discussion

### Catalyst characterization results

**Fig. 1:**
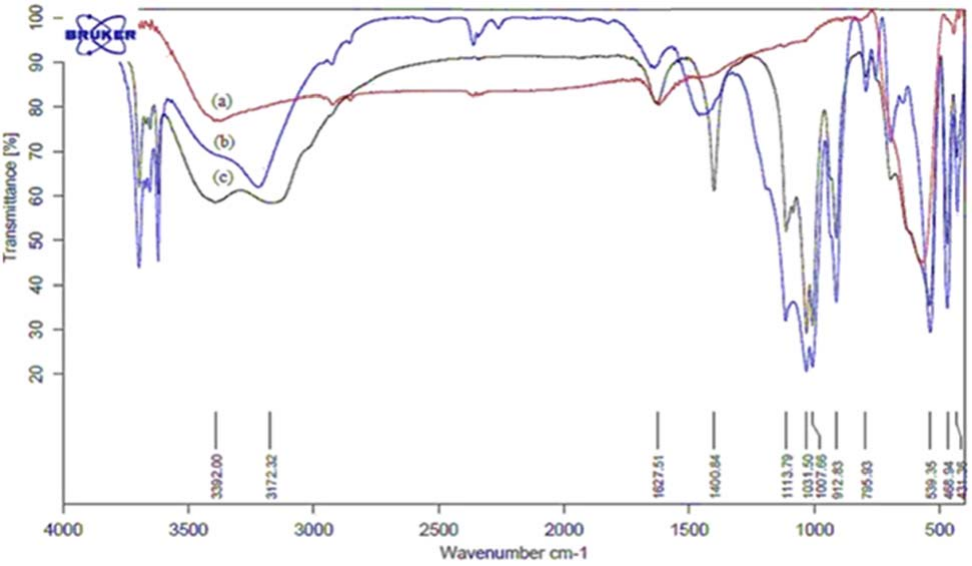
FT-IR spectra of: (a) Fe_3_O_4_, (b) nano cellulose/Ti^(IV)^ and (c) nano-cellulose/Ti^(IV)^/Fe_3_O_4_ nanoparticles.

The structural properties of catalyst nano-cellulose/Ti^(IV)^/Fe_3_O_4_ was determined by FT-IR, XRD, FESEM, TEM, TG-DTA, EDX, BET analysis and VSM analysis. Analysis (FT-IR) was performed to investigate the structure of the catalyst and to identify the positions of Lewis acid on the surface of the catalyst. The infrared spectra of (a) Fe_3_O_4_, (b) nano cellulose/Ti^(IV)^, and (c) nano-cellulose/Ti^(IV)^/Fe_3_O_4_ nano-particles are shown in Fig. 1. As the figure shows, in all spectra, the signal in 3100–3500 cm^−1^ indicates the stretching vibration of O–H; the signal in 1100 cm^−1^ indicate the vibration C–O band. In the nano-cellulose/Ti^(IV)^/Fe_3_O_4_ infrared spectrum, the bands of 539 cm^−1^, 1031 cm^−1^, and 3392 cm^−1^ are related to the vibrations of Fe–O, C–O and O–H, respectively.

**Fig. 2:**
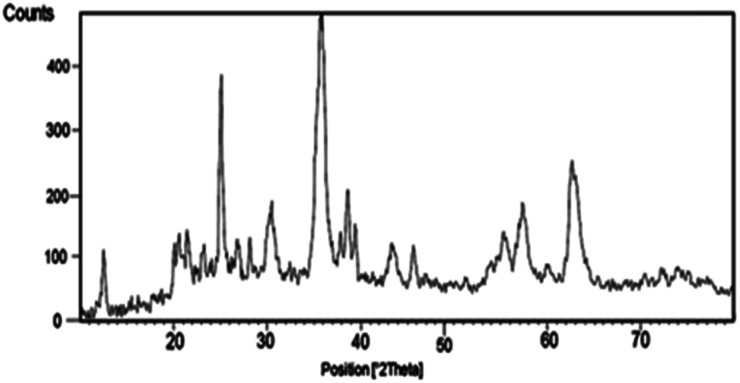
XRD patterns of nano-cellulose/Ti^(IV)^/Fe_3_O_4_.

The XRD pattern of the nano-cellulose/Ti^(IV)^/Fe_3_O_4_ catalyst is shown in Fig. 2. The broad peak of 2*θ* at 20–21 indicate the presence of cellulose. The observed peak 12, 30, 35, 37, 44, 57, 64 indicate the presence of Fe_3_O_4_ moiety in catalyst. The signals at 25, 38, 49, 55 and 63 are similar to the XRD signals for TiO_2_. These observed signals approve the structure of nano-cellulose/Ti^(IV)^/Fe_3_O_4_.

**Fig. 3:**
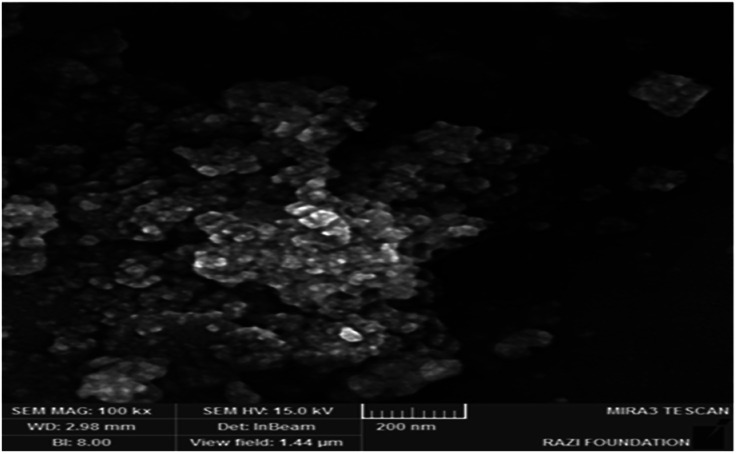
FESEM image of nano-cellulose/Ti^(IV)^/Fe_3_O_4_.

The morphology of the catalyst particles was determined by scanning electron microscopy. The FESEM image of the nano-cellulose/Ti^(IV)^/Fe_3_O_4_ catalyst is shown in Fig. 3. The spherical shape of the nano-particles is clearly visible in the image. In addition, the particle size in the FESEM model was about 25 nm.

**Fig. 4:**
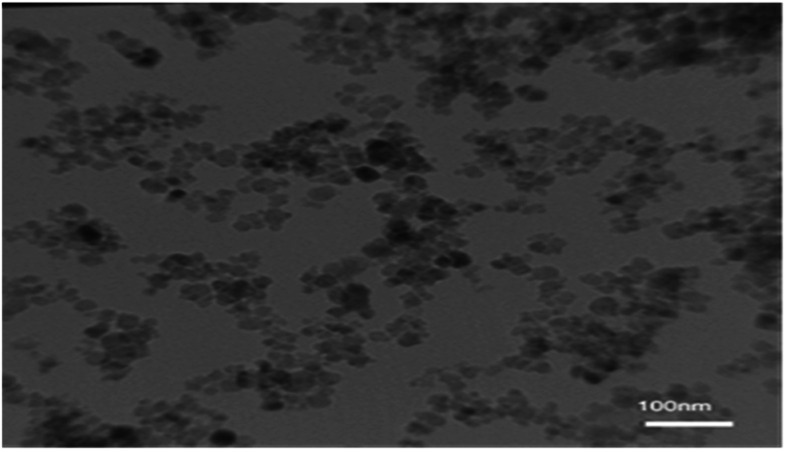
TEM image of nano-cellulose/Ti^(IV)^/Fe_3_O_4_.

The TEM image of the nano-cellulose/Ti^(IV)^/Fe_3_O_4_ catalyst is shown in Fig. 4. By using this technique, the average size of the catalyst is obtained, which shows the size of nano-particles at about 25 nm.

By thermal gravimetric analysis, the mass change of the catalyst can be measured as a function of temperature in the scanned state, or as a function of time in the isothermal state.

**Fig. 5:**
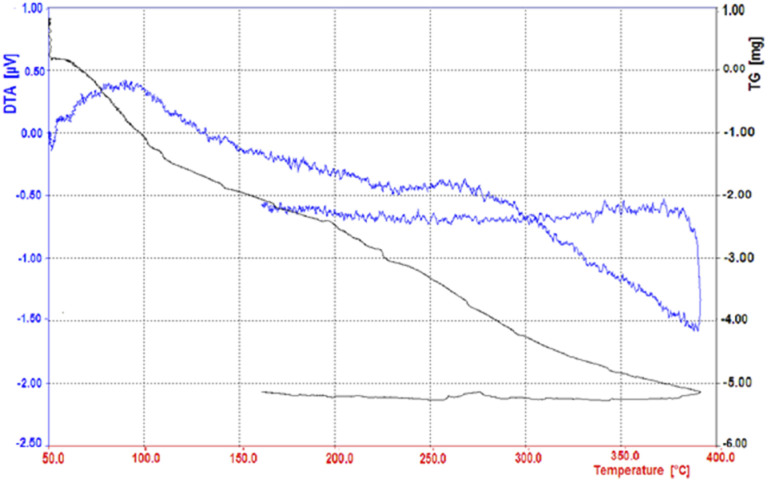
Thermal gravimetric analysis pattern of nano-cellulose/Ti^(IV)^/Fe_3_O_4_ catalyst.

Thermal changes associated with catalyst mass change such as decomposition, sublimation, reduction, adsorption and evaporation are measured in TGA. The thermal gravimetric decomposition pattern of the nano-cellulose/Ti^(IV)^/Fe_3_O_4_ catalyst from 50 to 400 °C is shown in Fig. 5. As shown in the figure, the nano-cellulose/Ti^(IV)^/Fe_3_O_4_ catalyst at 100 °C shows only 5% weight loss due to moisture loss. The catalyst is also stable at temperatures above 100 °C and can be used in high temperature organic reactions.

**Fig. 6:**
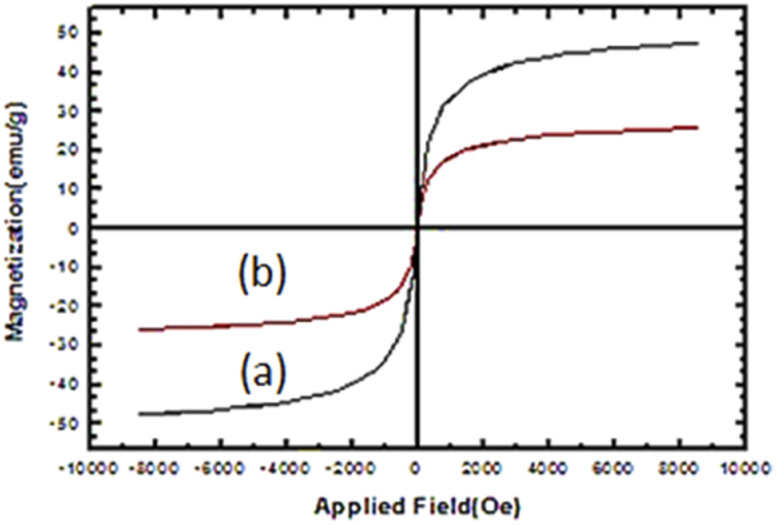
VSM images of (a) Fe_3_O_4_ and (b) nano-cellulose/Ti^(IV)^/Fe_3_O_4_.

VSM of Fe_3_O_4_ and nano-cellulose/Ti^(IV)^/Fe_3_O_4_ at room temperature are shown in Fig. 6. The decrease in the magnetic saturation of the nano-cellulose/Ti^(IV)^/Fe_3_O_4_ catalyst compared to Fe_3_O_4_ is due to the presence of non-magnetic material (cellulose) with Fe_3_O_4_ nano-particles. Despite this reduction in magnetic saturation, the catalyst can still be separated from the solution by an external magnetic field.

In this study, cellulose/Ti^(IV)^/Fe_3_O_4_ catalyst was applied for synthesis of chromenes *via* reaction of aromatic aldehydes, malononitrile and β-diketone. In order to determine the best conditions for the reaction, first the multicomponent reaction of benzaldehyde (1 mmol), dimedone (1 mmol) and malononitrile (1.1 mmol) was selected as the model reaction. Progression of the reaction was followed by thin layer chromatography (TLC). This reaction was performed for different conditions; the results are shown in Table 1. The results of Table 1 show that the optimum amount of nano-cellulose/Ti^(IV)^/Fe_3_O_4_ catalyst with this method is 0.05 g per 1 mmol of other materials. The optimum conditions for the reaction is solvent-free at 70 °C. According to Table 1, TiO_2_ can also promote the reaction well, but because it is not magnetic, its workup is more difficult. Meanwhile, cellulose and cellulose/Fe_3_O_4_ can not promote the reaction well.

**Table 1:** Optimization of reaction conditions for the preparation of chromene 4a^a^

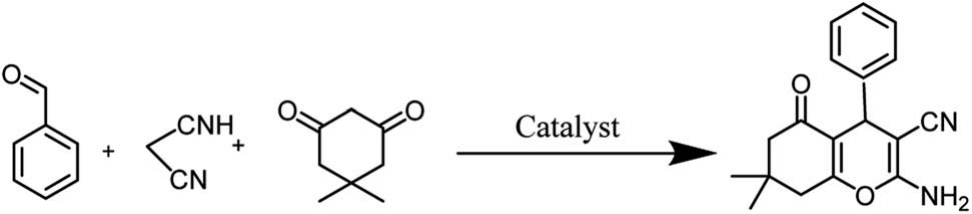					
Row	Catalyst (g)	*T* (°C)	Solvent	Time (min)	Yield^c^ (%)
1	0.1^b^	R.T.	Ethanol	15	90
2	0.1^b^	R.T.	CH_2_Cl_2_	15	80
3	0.1^b^	70	—	50	99
4	0.05^b^	70	—	50	95
5	0.04^b^	70	—	50	90
6	TiO_2_ (0.05)	70	—	50	97
7	Cellulose (0.05)	70	—	50	40
8	Cellulose/Fe_3_O_4_ (0.05)	70	—	50	65

a Benzaldehyde (1 mmol), dimedone (1 mmol), malononitrile (1.1 mmol) and solvent (2 mL).

b Nano-cellulose/Ti^(IV)^/Fe_3_O_4._

c Isolated yield.

According to modified conditions, a number of chromenes derivatives were prepared in the presence of 0.05 g of nano-cellulose/Ti^(IV)^/Fe_3_O_4_ catalyst under solvent free at 70 °C (Table 2). In all of these studied examples, the aldehyde derivatives bearing either electron donating or electron withdrawing substituent reacted smoothly to give the corresponding chromenes in excellent yields.

**Table 2:** Nano-cellulose/Ti^(IV)^/Fe_3_O_4_ catalyzed synthesis of chromens under solvent-free condition^a^^,^^b^

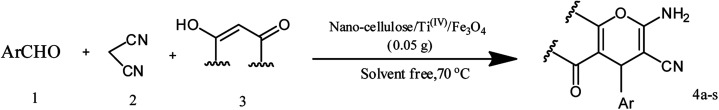					
Entry	Compound with an active acidic H–C	Aldehyde	Product	Time (min)/yield^b^ (%)	M.P. °C (Obs.) M.P. (lit.) [Ref.]
1	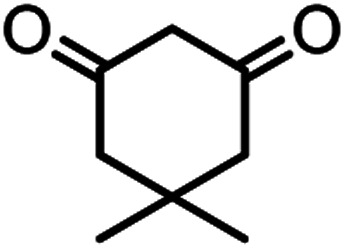	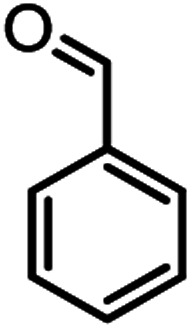	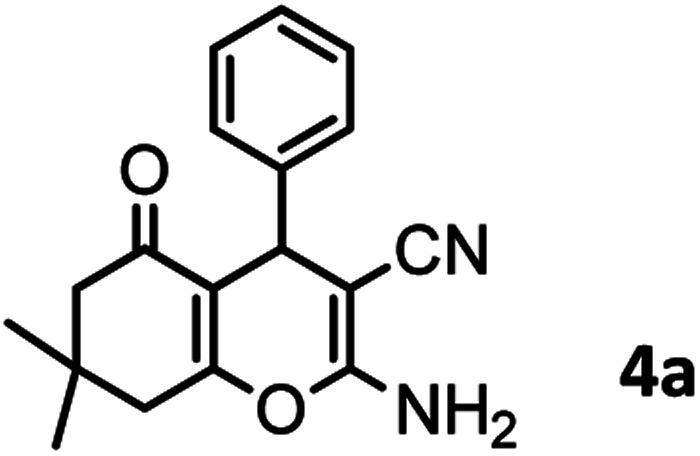	50/95	238–240 (237–239)^8^
2	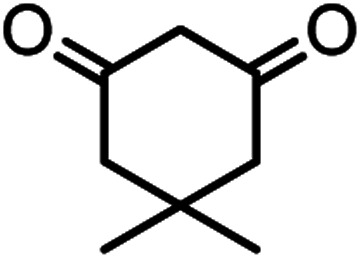	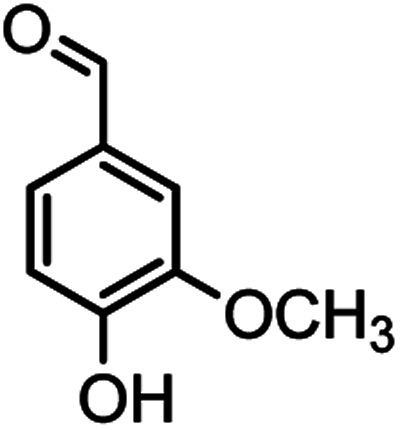	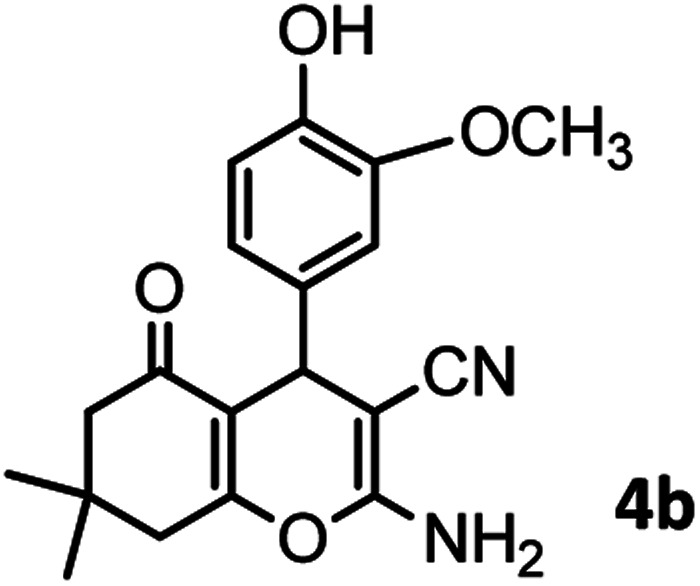	40/82	230–231 (227–229)^8^
3	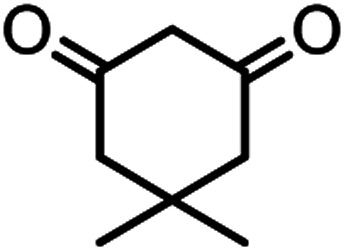	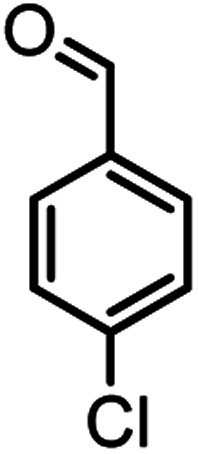	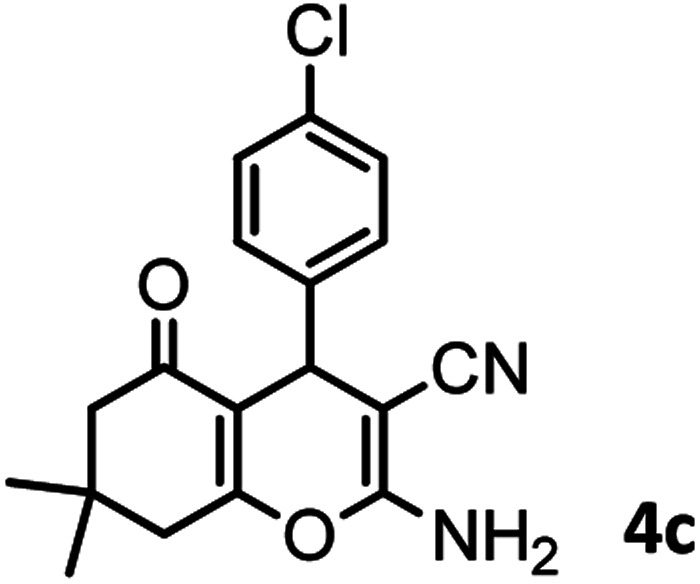	38/88	210–212 (213–214)^9^
4	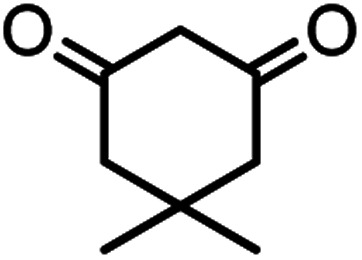	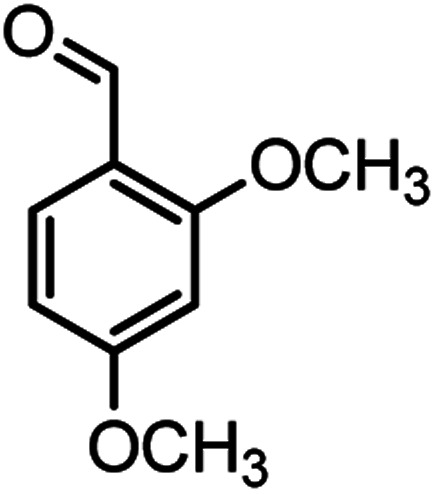	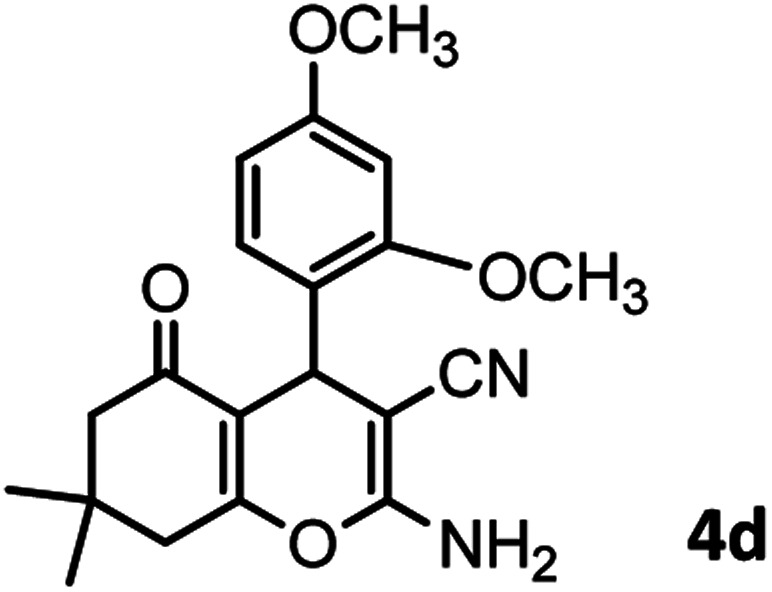	53/88	229–231 (227–229)^9^
5	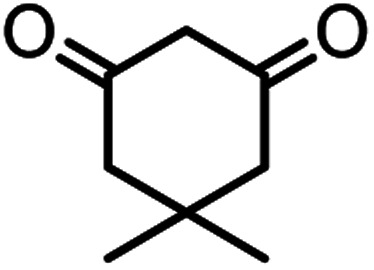	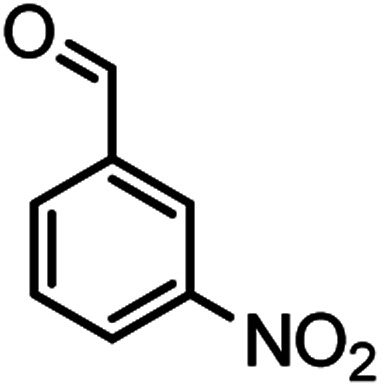	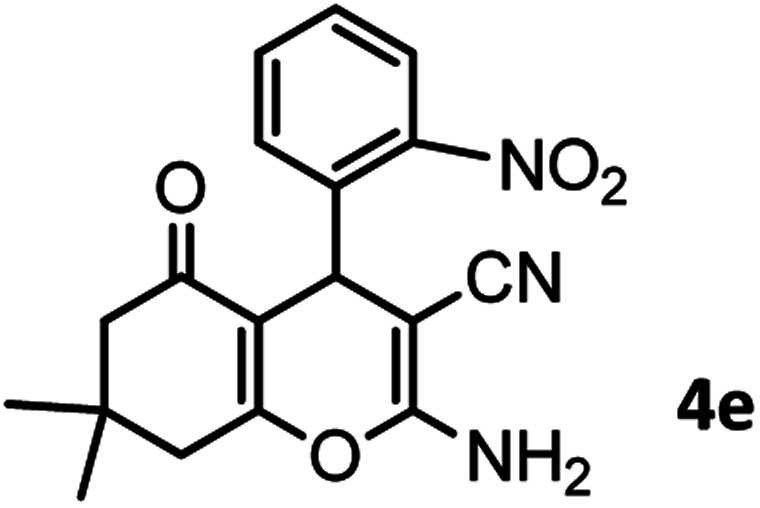	55/79	236–238 (238–239)^10^
6	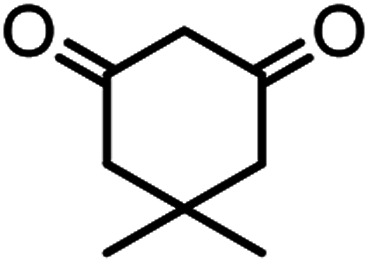	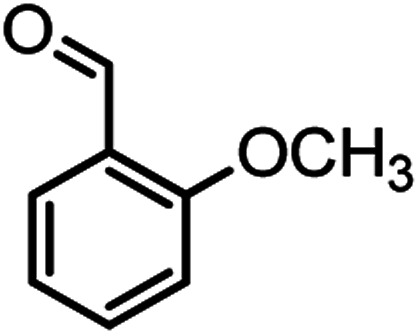	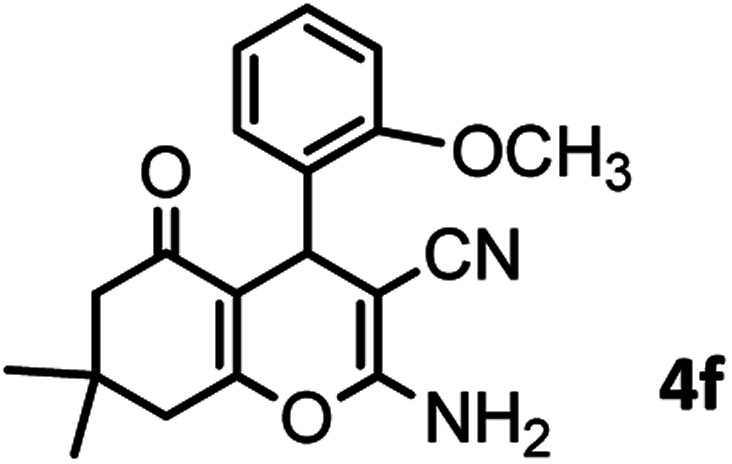	44/82	195–197 (196–198)^9^
7	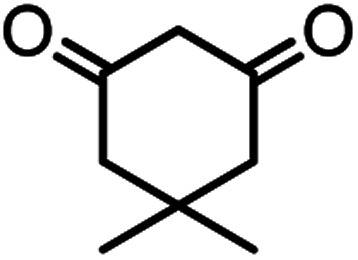	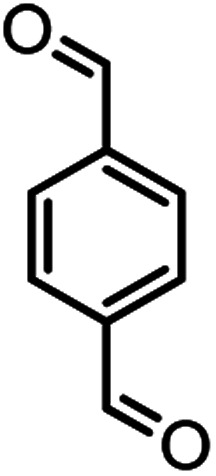	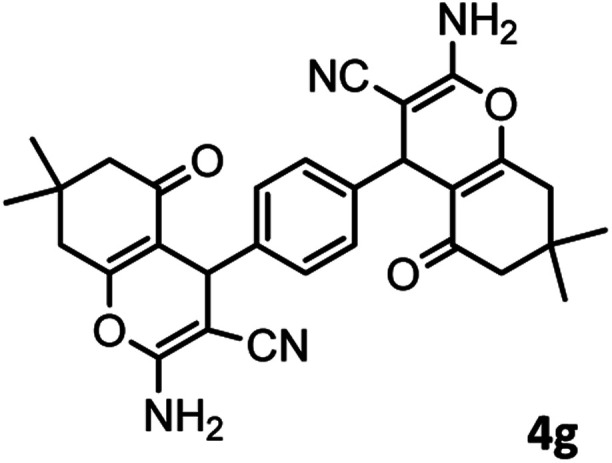	59/87	>280
8	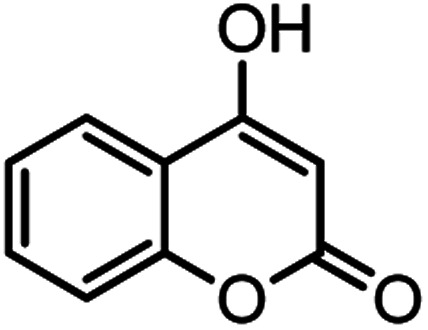	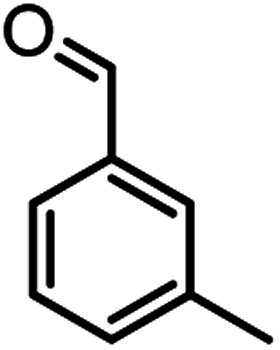	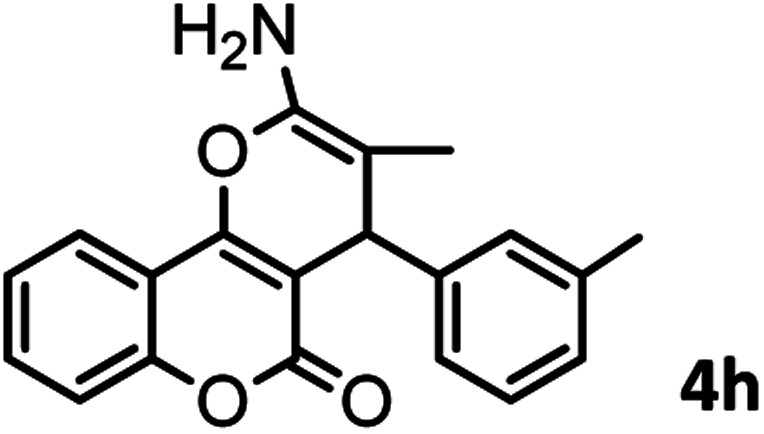	37/84	253–255 (254–255)^9^
9	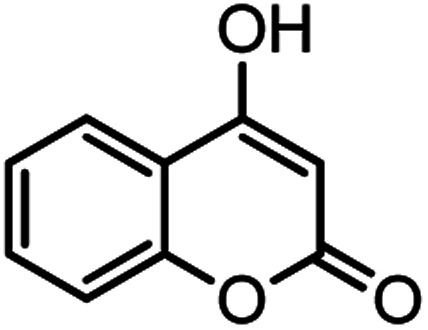	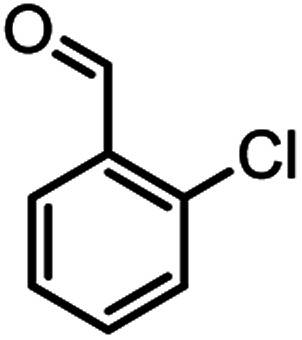	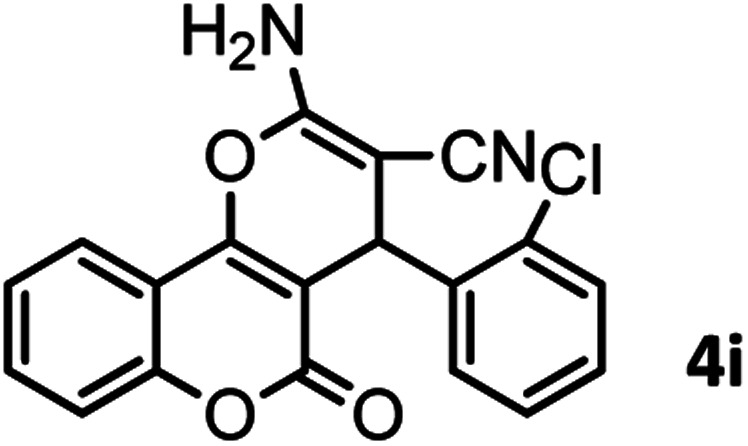	40/85	265–267 (266–268)^11^
10	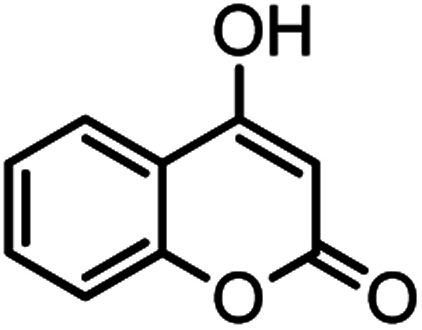	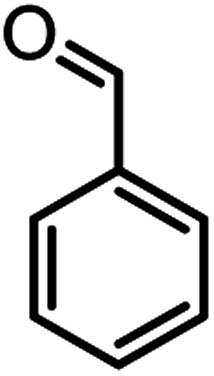	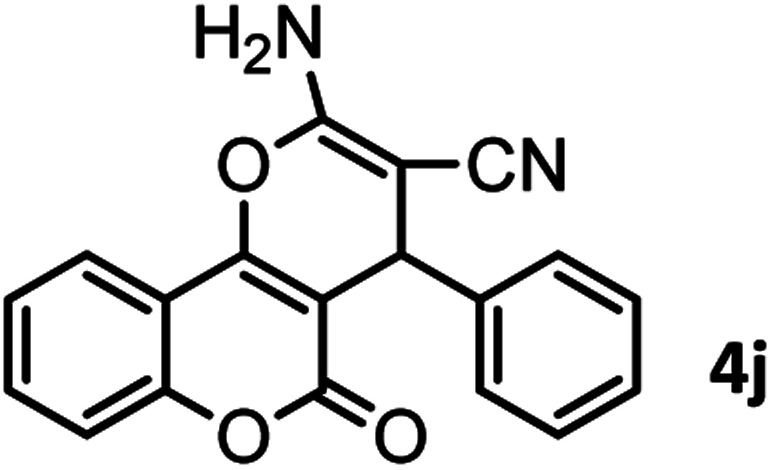	52/87	259–261 (256–258)^12^
11	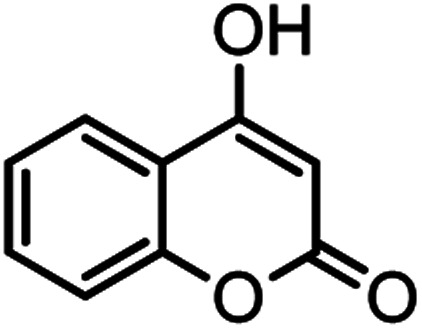	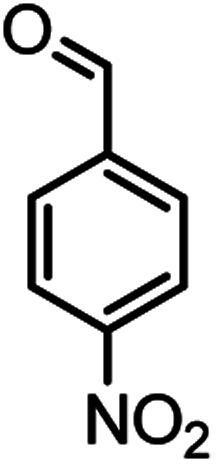	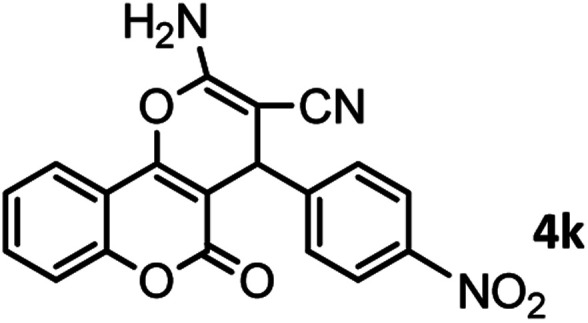	36/80	252–254 (250–252)^13^
12	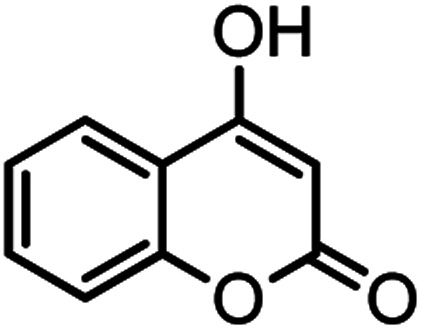	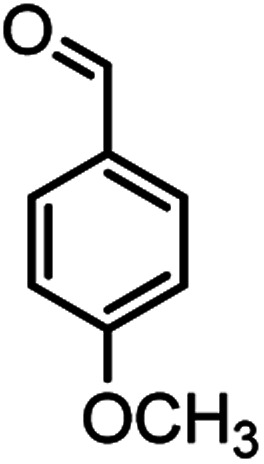	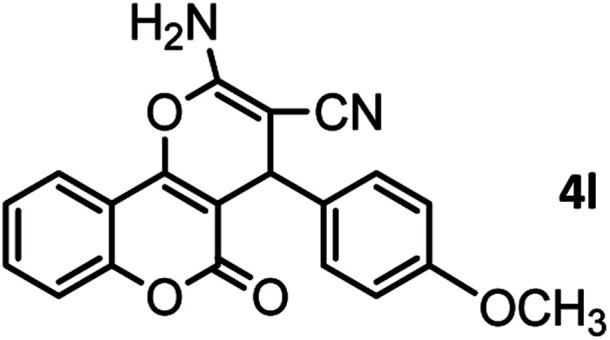	46/84	246–248 (247–249)^13^
13	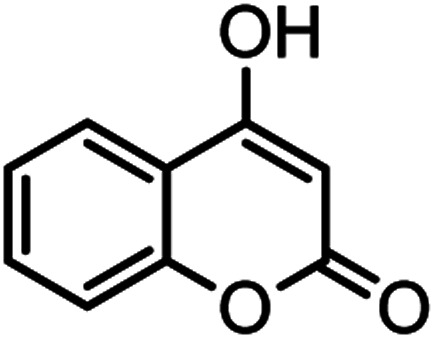	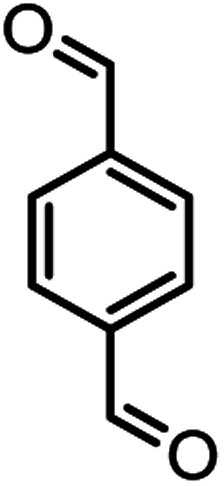	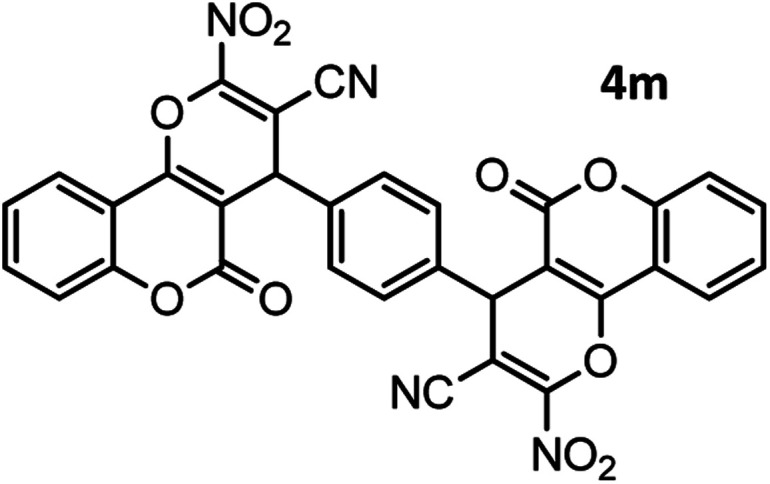	44/81	>280
14	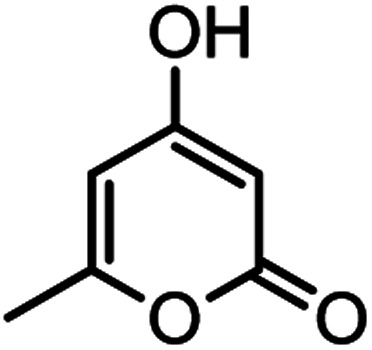	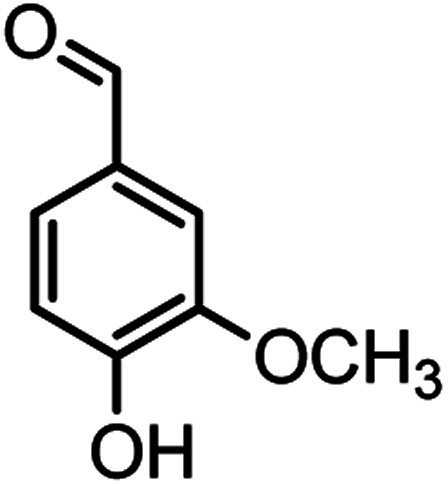	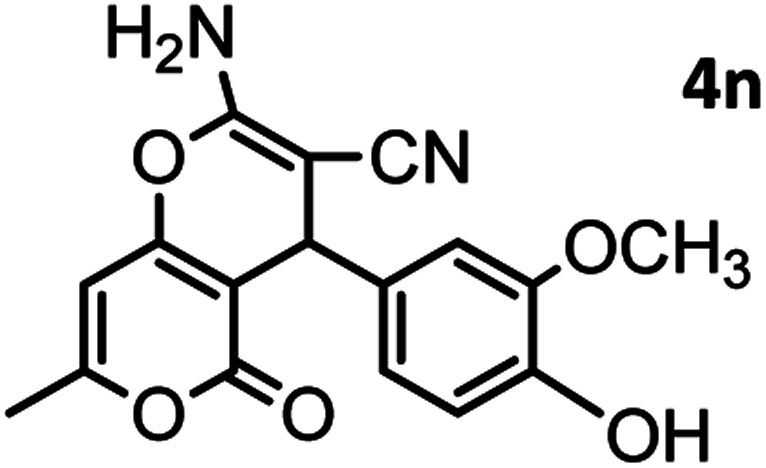	44/85	260–262 (258–259)^14^
15	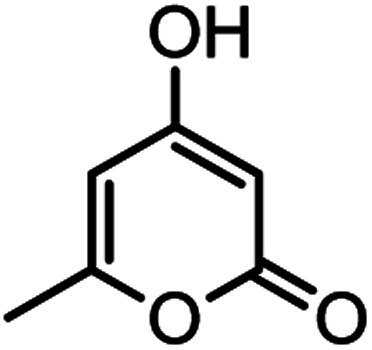	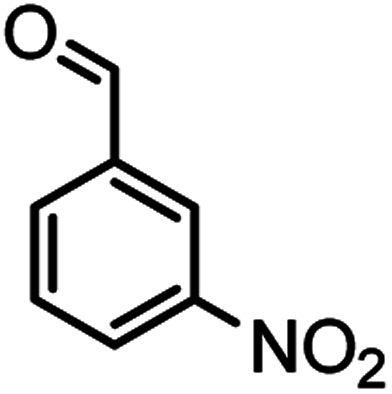	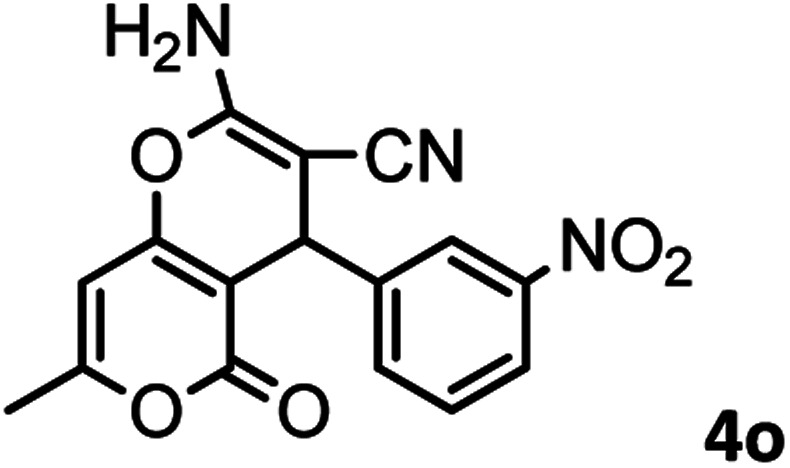	54/86	236–238 (235–237)^8^
16	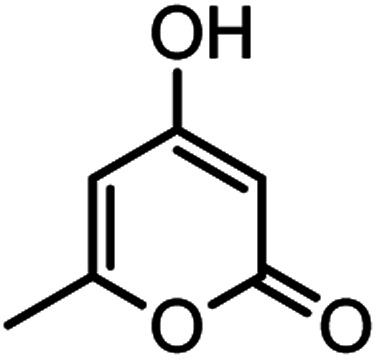	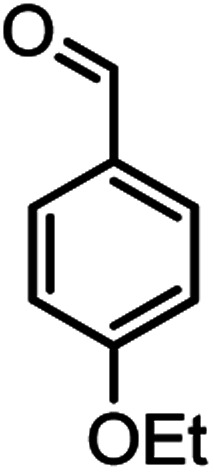	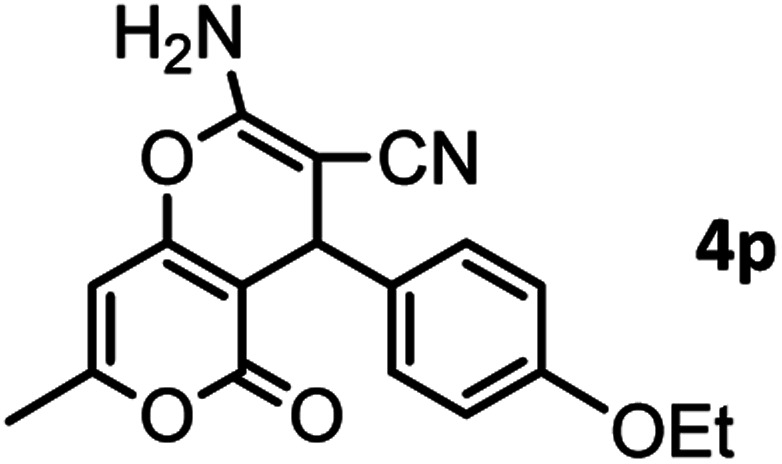	37/88	230–232 (233–235)^15^
17	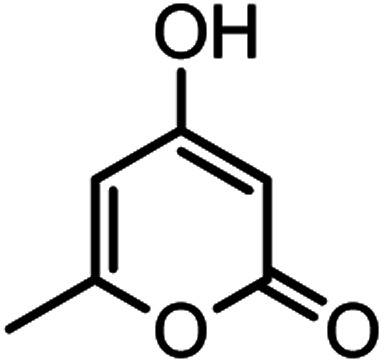	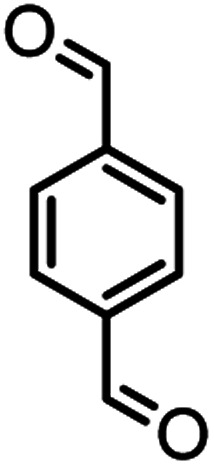	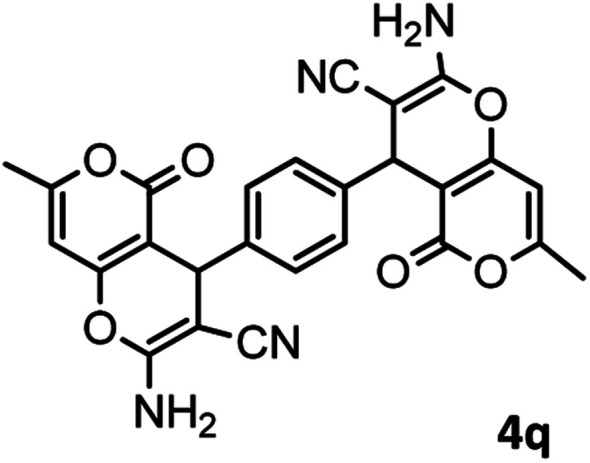	53/88	>280
18	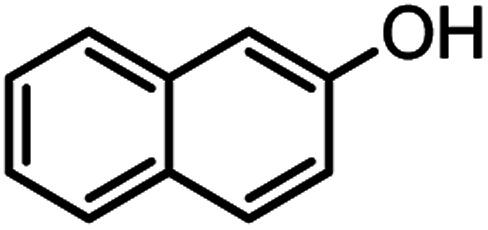	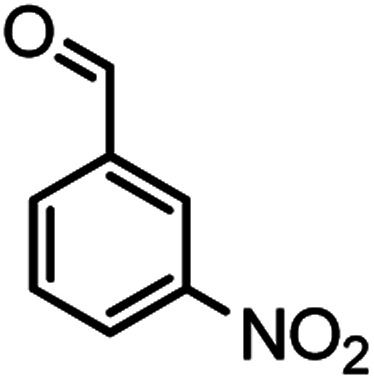	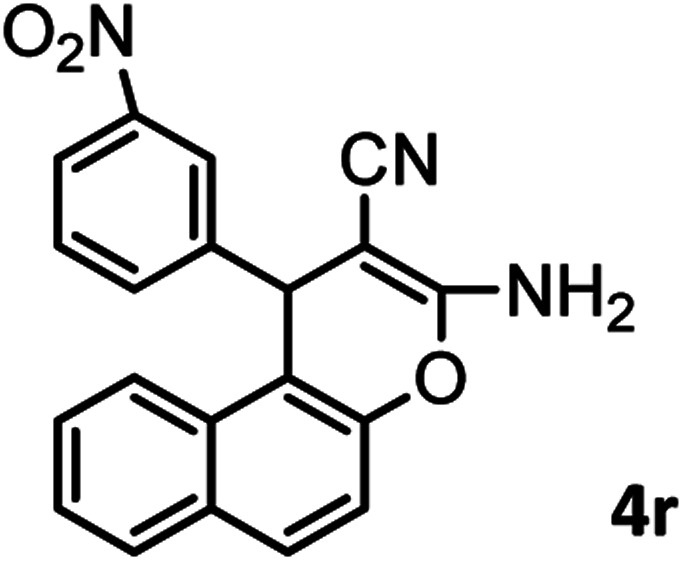	56/86	238–240 (239–241)^16^
19	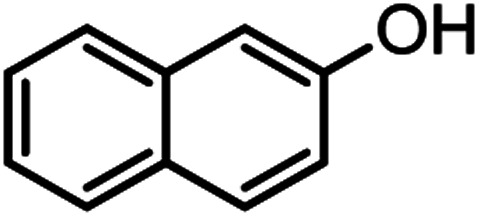	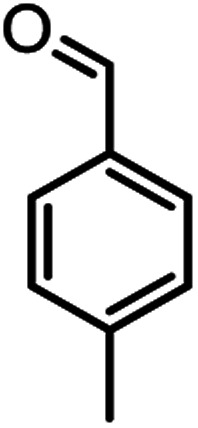	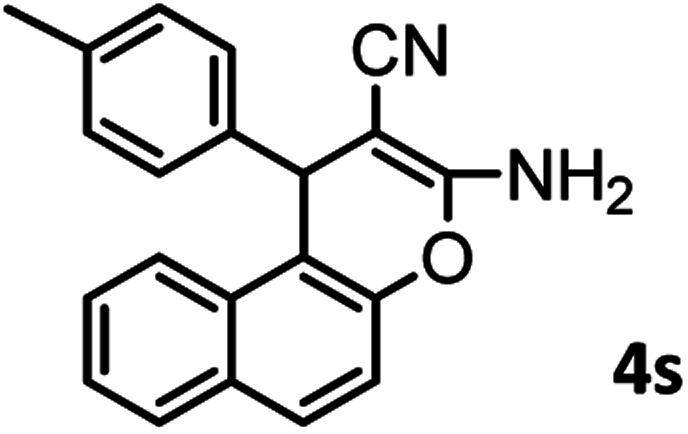	48/88	268–270 (271–272)^16^
20	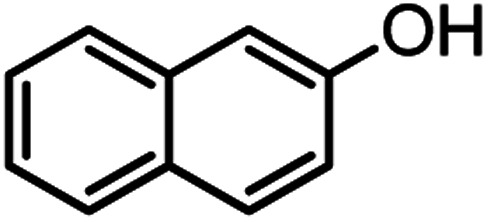	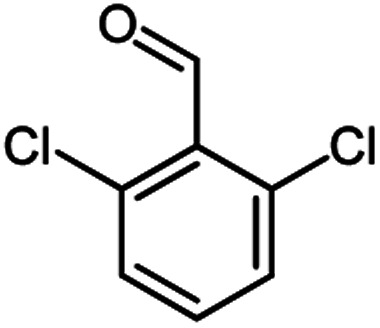	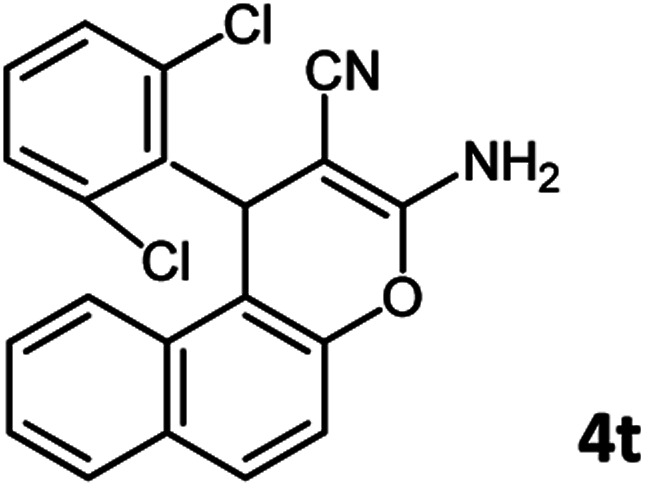	180/80	200–202

a Aldehyde (1 mmol), 1,3-diketone (1 mmol), malononitrile (1.1 mmol).

b Isolated yield.

Using 2,6-dichlorobenzaldehyde as an aldehyde with steric hindrance, caused addition in time and reduction in yield of reaction (Table 2, entry 20).

**Fig. 7:**
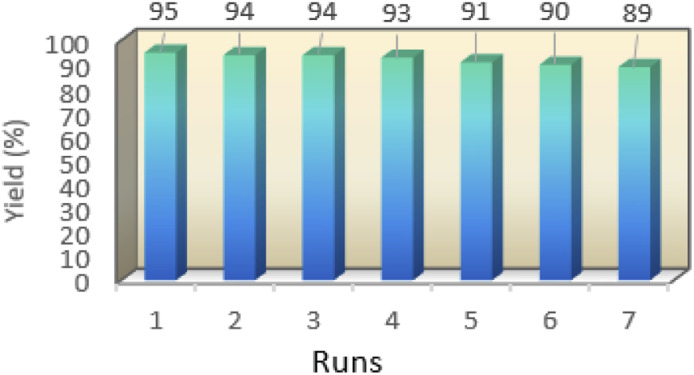
The reusability experiment of nano-cellulose/Ti^(IV)^/Fe_3_O_4_.

One of the important features of solid acid catalysts is their reusability. The results showed that the catalyst was reused 7 times in the preparation of 4a with low reduction in activity. The results are shown in Fig. 7. The best number run for reusability is 5 run with 4% reduction in yield of 4a.

The efficiency of nano-cellulose/Ti^(IV)^/Fe_3_O_4_ catalyst in the synthesis of 4a was compared with other reported catalysts and the data were shown in Table 3. According to these results, our catalyst is a suitable catalyst with high efficiency.

**Table 3:** Comparison the efficiency of the nano-cellulose/Ti^(IV)^/Fe_3_O_4_ catalyst with other reported catalysts in synthesis of 2-amino-7,7-dimethyl-5-oxo-4-phenyl-5,6,7,8-tetrahydro-4*H*-chromene-3-carbonitrile (4a)^a^

Entry	Catalyst	Time	Condition	Yield (%)	Ref.
1	Zn(l-proline)_2_ (20 mol%)	3 h	Reflux, EtOH	85	17
2	ChCl/urea/thiourea (36 mol%)	22 min	Solvent-free,100 °C	90	18
3	[Co(MCG)(H_2_O)_3_] (5 mol%)	20 min	Reflux/H_2_O : EtOH (1 : 1)	88	19
4	SiO_2_ (0.03 g)	4 min	US, C_2_H_5_OH	86	20
5	Triethanolamine (5 mol%)	1.5 h	EtOH, 80 °C	98	21
6	NiFe_2_O_4_ NPs (12 mg)	30 min	Reflux, EtOH	90	22
7	RTIL (2 mol%)	30 min	Reflux,ethanol	86	23
8	Bis-Su (10 mg)	35 min	H_2_O : EtOH (1 : 1), 80 °C	84	24
9	BaFe_12_O_19_@IM (5 mol%)	20 min	Reflux/ethanol	88	25
10	PANF-D (15 mol%)	60 min	Reflux	97	26
11	BAILs-ClO_4_ (5 mol%)	28 min	r.t., solvent-free	91	27
12	Ni@Fe-doped CeO_2_/chitosan (11 mg)	10 min	EtOH, 60 °C	90	28
13	TEA (25 mol%)	15 min	Reflux, EtOH	90	29
14	Nano-cellulose/TiCl_4_/Fe_3_O_4_ (0.05 g)	50 min	70 °C, solvent-free	95	This work

a RTIL: room-temperature ionic liquids: Bis-Su: 1,1′-(butane-1,4-diyl)bis(pyrrolidine-2,5-dione): PANF-D: polyacrylonitrile fiber.

### In preparation of 4a

**Scheme 1:**
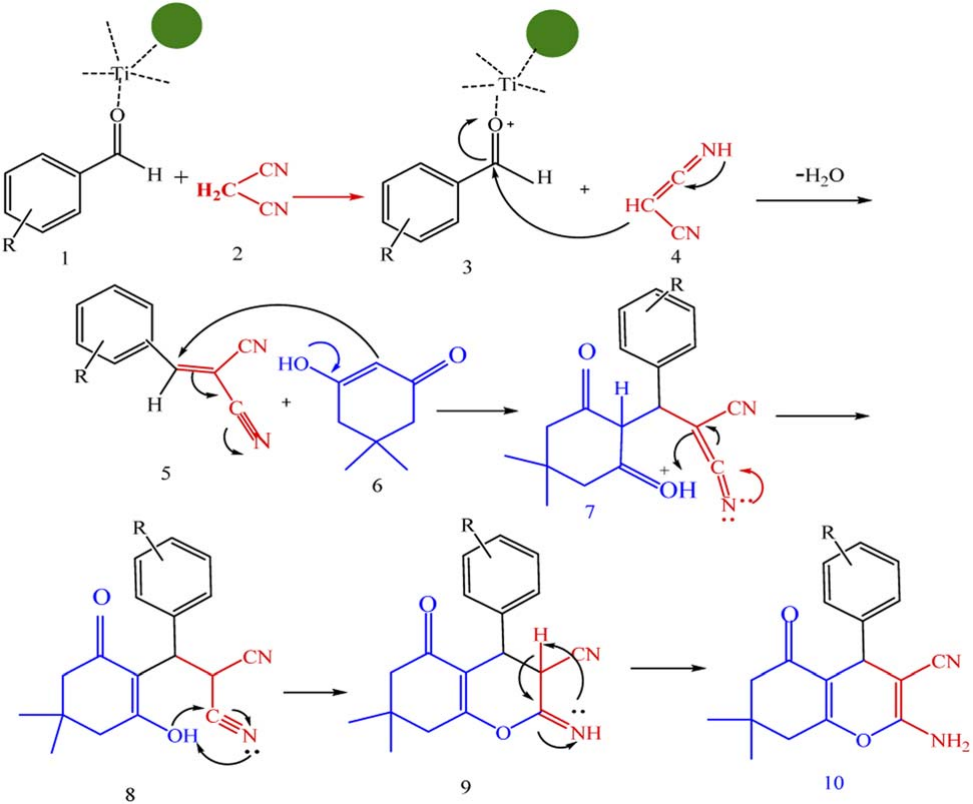
A plausible mechanism for synthesis of chromens in the presence of nano-cellulose/Ti^(IV)^/Fe_3_O_4_.

A plausible mechanism for synthesis of chromens in the presence of nano-cellulose/Ti^(IV)^/Fe_3_O_4_ is shown in Scheme 1. According to this mechanism, the titanium section of catalyst actives the carbonyl group of aldehyde (1) to react with malononitrile (2) to form condensation product (5). The enol form of dimedone is added to 5*via* Michael addition to form intermediate 7. By internal proton transfer, the intermediate 8 and then by cyclization, the intermediate 9 is formed. The product 10 is produced form 9*via* tautomerization.

## Conclusion

We have described the synthesis of nano-cellulose/Ti^(IV)^/Fe_3_O_4_ as a core–shell nano-catalyst which was used for the one-pot synthesis of various chromens at 70 °C under solvent-free conditions. Due to simple preparation heterogeneous nature of the catalyst, solvent free mild reaction conditions, higher yields, and recovery capability, it can be concluded that nano-cellulose/Ti^(IV)^/Fe_3_O_4_ shows very high activity. The catalyst is reused several times, while generally maintaining the same reaction yield.

## Conflicts of interest

There are no conflicts to declare.

## Supplementary Material

RA-012-D2RA05057A-s001
