# Supplementary material for: One-pot synthesis of chromenes in the presence of nano-cellulose/Ti(IV)/Fe3O4 as natural-based magnetic nano-catalysts under solvent free conditions
Source: RSC Adv. 2022 Sep 28;12(42):27555–63. doi: 10.1039/d2ra05057a (PMC9517711; doi:10.1039/d2ra05057a)

Electronic Supplementary Material (ESI) for RSC Advances.
This journal is © The Royal Society of Chemistry 2022

Supplementary

One-pot synthesis of chromenes in the presence of nano-cellulose/Ti$^{(IV)}$/Fe_3O_4 as natural based magnetic nano-catalysts under solvent free condition

Raziyeh Gholami[1] ,Abdolhamid Bamoniri*[1], Bi Bi Fatemeh Mirjalili,[2]
[1]*Department of Organic Chemistry, Faculty of Chemistry, University of Kashan, Kashan, I.R. Iran.*
**Corresponding authors: Tel.: +98-31-55912384; E-mail: bamoniri@kashanu.ac.ir*
[2]*Department of Chemistry, Faculty of Science, Yazd University, I.R.Iran*

Physical and spectral data

2-amino-7,7-dimethyl-5-oxo-4-phenyl-5,6,7,8-tetrahydro-4*H*-chromene-3-carbonitrile

FT-IR [KBr ῡ (cm^{-1})]: 3395, 3324 (NH_2), 3083, 3028 (C_{ArH}), 2198 (C≡N), 1661 (C=O), 1602 (C=C), 1035 (C-O).

^{1}H NMR (400 MHz, DMSO-d_6) δppm: 0.94 (s, 3H, CH_3), 1.02 (s, 3H, CH_3), 2.08 (m, 2H, CH_2), 2.24 (m, 2H, CH_2), 4.15 (s, 1H, CH), 6.97 (s, 2H, NH_2), 7.14 (t, *J* = 7.12 Hz, 3H, Ar-H), 7.25-7.28 (m, 2H, Ar-H) .

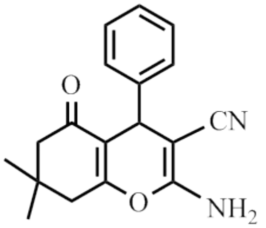

FT-IR

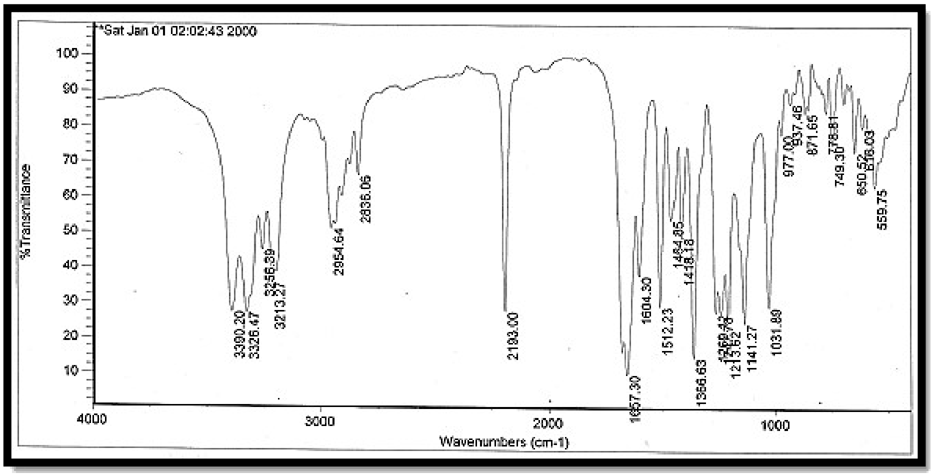

^{1}H NMR

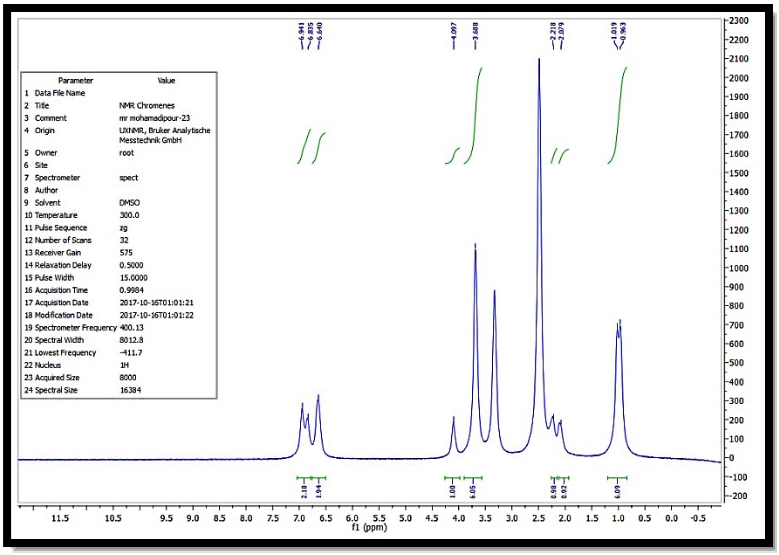

2-amino-4-(4-hydroxy-3-methoxyphenyl)-7,7-dimethyl-5-oxo-5,6,7,8 tetrahydro-4*H*-chromene-3-carbonitrile

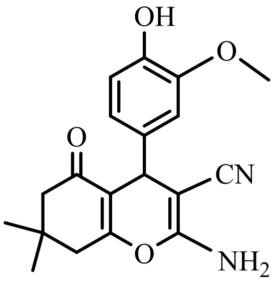

FT-IR [KBr ῡ (cm^{-1})]: 3497 (O-H), 3403, 3324 (NH_2), 3016 (Ar-H), 2192 (C≡N), 1654 (C=O), 1603 (C=C), 1034 (C-O).
^{1}H NMR (400 MHz, DMSO-d$_6$) δppm: 0.95 (s, 3H, CH_3), 1.02 (s, 3H, CH_3), 2.08 (m, 2H, CH_2), 2.23 (m, 2H, CH_2), 3.69 (s, 3H, OCH_3), 4.05 (s, 1H, CH), 6.48-6.51 (m, 1H), 6.62-6.66 (m, 2H), 6.89 (s, 2H, NH_2), 8.80 (s, 1H, OH).

x

FT-IR

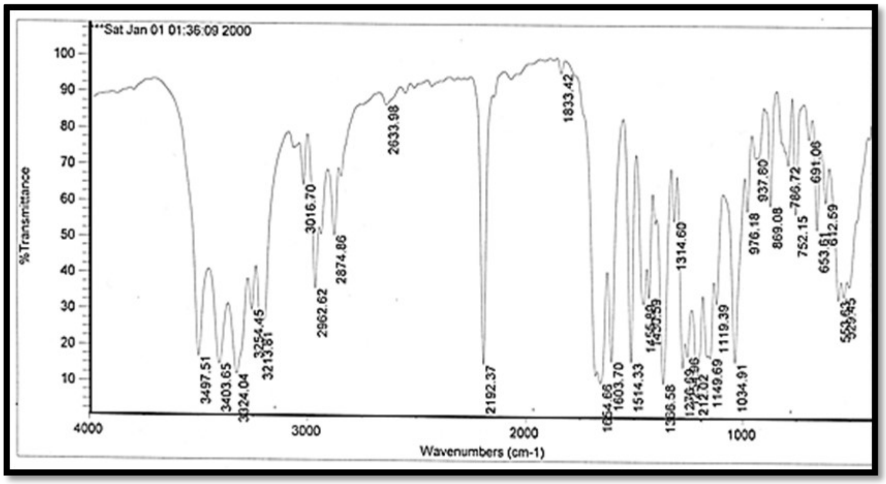

***Sat Jan 01 01:36:09 2000
%Transmittance
Wavenumbers (cm-1)
3497.51
3403.65
3324.04
3254.45
3213.81
3016.70
2962.62
2874.86
2633.98
2192.37
1833.42
1603.70
1514.33
1336.58
1314.60
1119.39
1149.69
1034.91
976.18
937.80
869.08
786.72
752.15
691.06
612.59
529.45

^{1}H NMR

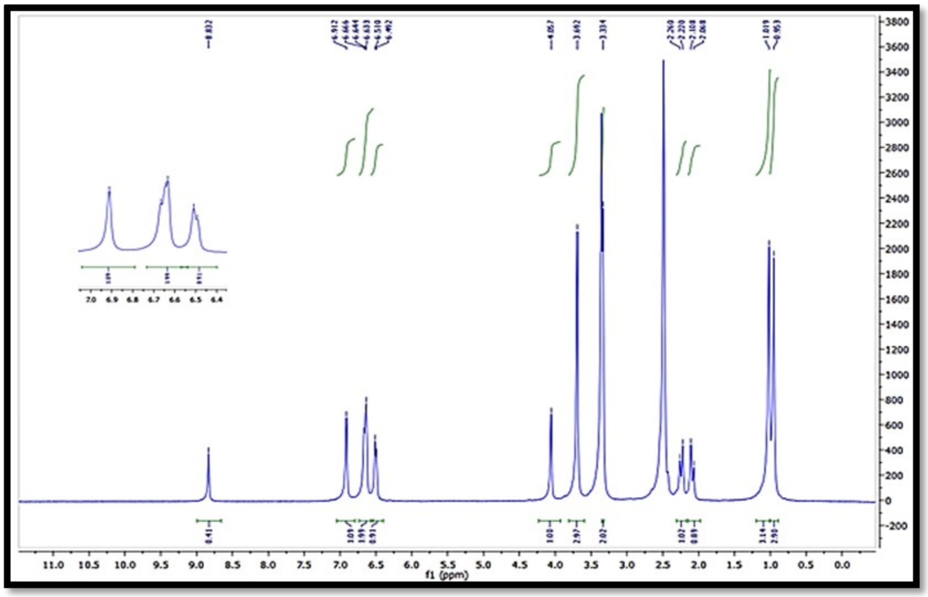

f1 (ppm)

2-amino-4-(4-chlorophenyl)-7,7-dimethyl-5-oxo-5,6,7,8-tetrahydro-4*H* chromene-3-carbonitrile

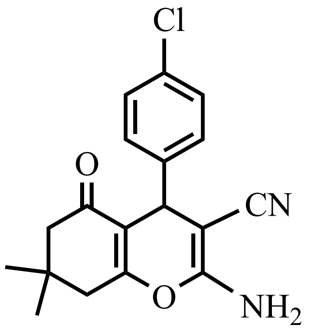

FT-IR [KBr ῡ (cm^{-1})]: 3380, 3323 (NH_2), 3183, 2959 (Ar-H), 2188 (C≡N), 1676 (C=O), 1603 (C=C), 1032 (C-O).
^{1}H NMR (400 MHz, DMSO-d_6) δppm: 0.92 (s, 3H, CH_3), 1.01 (s, 3H, CH_3), 2.08 (m, 2H, CH_2), 2.22 (m, 2H, CH_2), 4.17 (s, 1H, CH), 7.03 (s, 2H, NH_2), 7.15 (d, *J* = 8.44 Hz, 2H), 7.32 (d, *J* = 8.4 Hz, 1H).

FT-IR

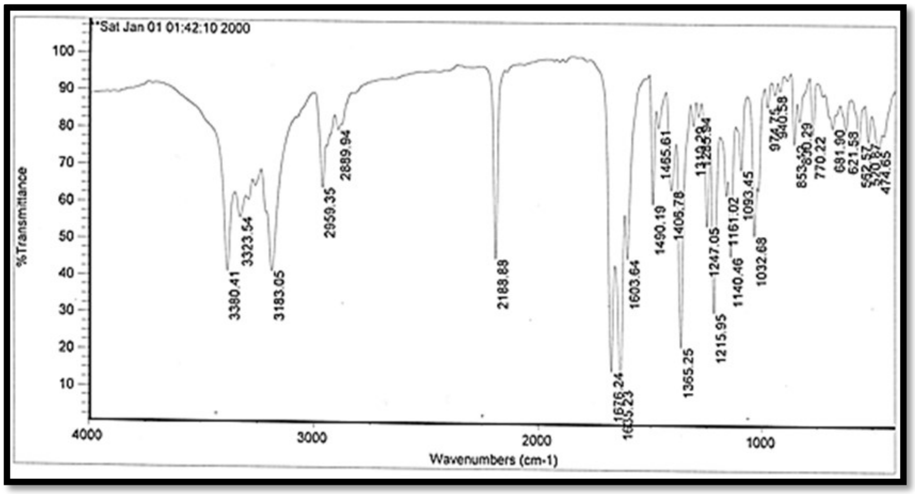

^{1}H NMR

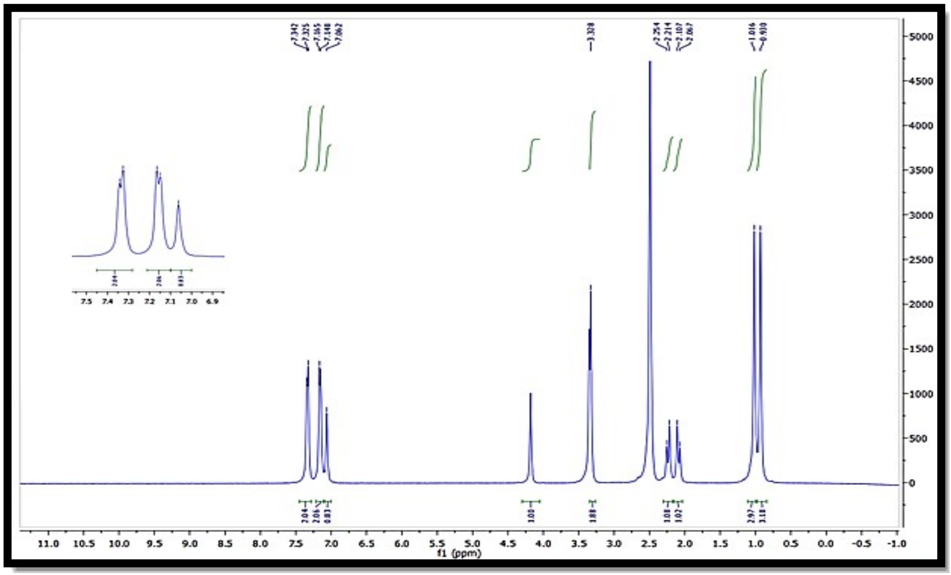

2-amino-4-(2,4-dimethoxyphenyl)-7,7-dimethyl-5-oxo-5,6,7,8-tetrahydro-4*H*-chromene-3-carbonitrile

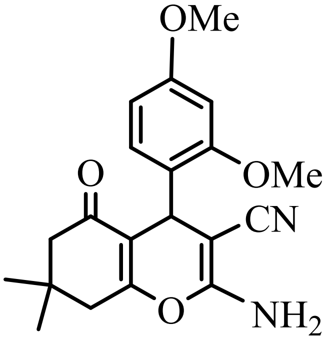

FT-IR [KBr ῡ (cm^{-1})]: 3390, 3326 (NH_2), 3256, 3213, 2954 (Ar-H), 2193 (C≡N), 1657 (C=O), 1604 (C=C), 1031 (C-O).

^{1}H NMR (400 MHz, DMSO-d_6) δppm: 0.96 (3H, s, CH_3), 1.01 (3H, s, CH_3), 2.07 (m, 2H, CH_2), 2.21 (m, 2H, CH_2), 3.68 (6H, s, OCH_3), 4.09 (1H, s, CH), 6.83 (4H, br s, H-Ar, NH_2).

FT-IR

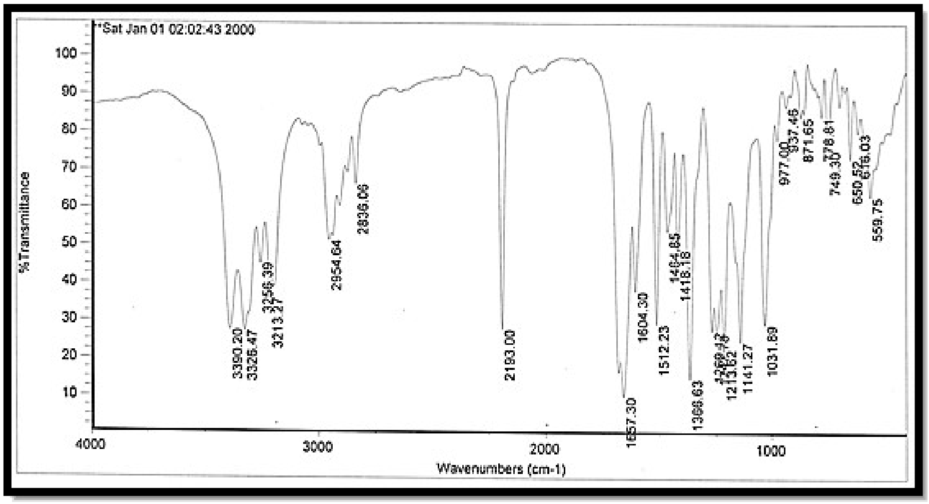
*Sat Jan 01 02:02:43 2000
%Transmittance
Wavenumbers (cm-1)
3390.20
3326.47
3256.39
3213.27
2954.64
2836.06
2193.00
1657.30
1604.30
1512.23
1464.85
1418.18
1368.63
1213.62
1141.27
1031.89
977.00
937.46
871.65
778.81
749.30
650.52
616.03
559.75

^{1}H NMR

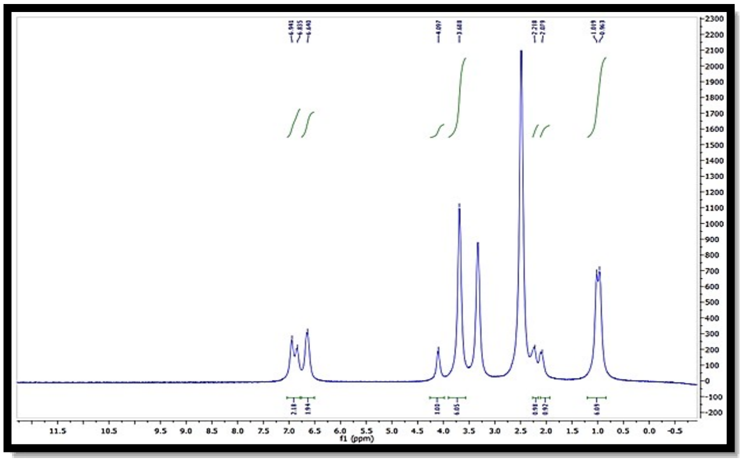
f1 (ppm)

2-amino-7,7-dimethyl-4-(2-nitrophenyl)-5-oxo-5,6,7,8-tetrahydro-4*H*-chromene-3-carbonitrile

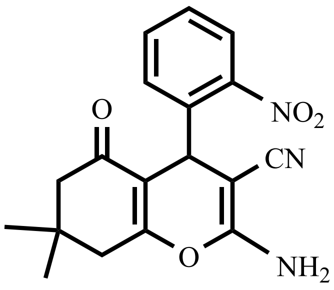

FT-IR [KBr ῡ (cm^{-1})]: 3471, 3332 (NH_2), 3255, 3210, 2960 (Ar-H), 2194 (C≡N), 1688 (C=O), 1602 (C=C), 1525, 1596 (NO_2), 1041 (C-O).

^{1}H NMR (400 MHz, DMSO-d$_6$) δppm: 0.81 (s, 3H, CH_3), 0.94 (s, 3H, CH_3), 1.94 (m, 2H, CH_2), 2.13 (m, 2H, CH_2), 4.86 (s, 1H), 7.11 (s, 2H, NH_2), 7.28 (dd, J = 6.78, 1.00 Hz, 1H), 7.36 (t, J = 7.2 Hz, 1H), 7.57-7.75 (m, 2H).

FT-IR

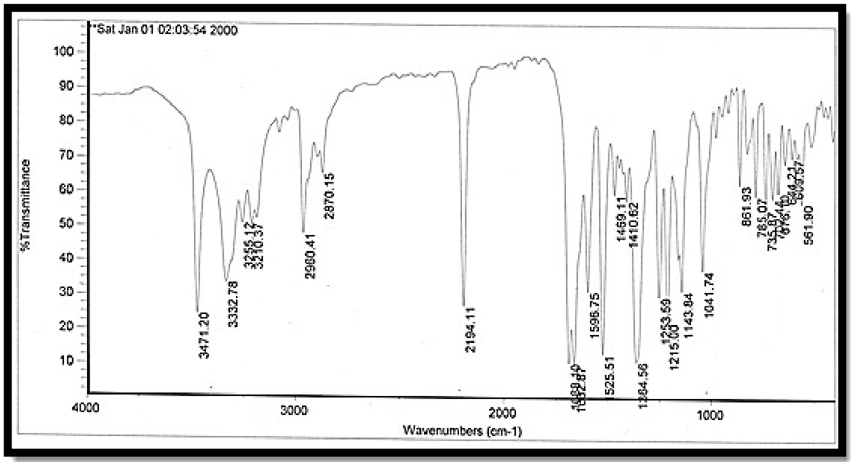

^{1}H NMR

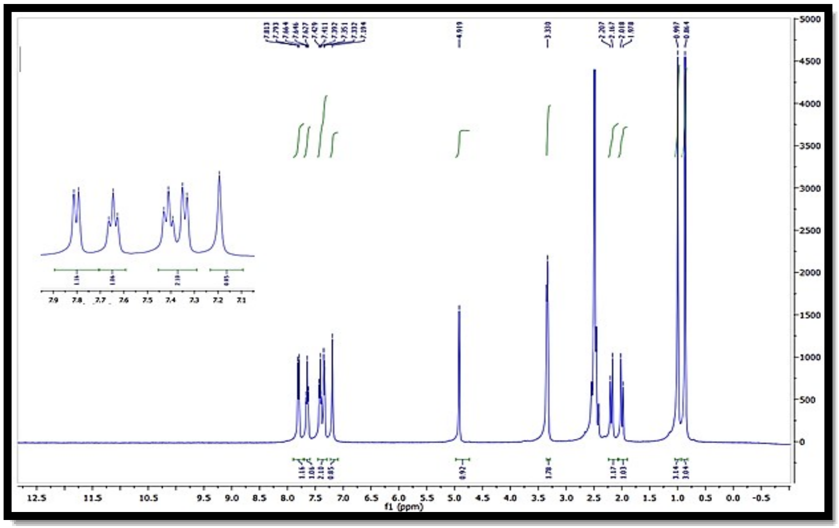

2-amino-4-(2-methoxyphenyl)-7,7-dimethyl-5-oxo-5,6,7,8-tetrahydro-4*H*-chromene-3-carbonitrile

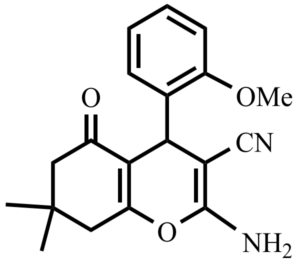

FT-IR [KBr ῡ (cm^{-1})]: 3396, 3329 (NH_2), 3262, 3219, 2964 (Ar-H), 2189 (C≡N), 1685 (C=O), 1654 (C=C), 1036 (C-O).
^{1}H NMR (400 MHz, DMSO-d_6) δppm: 0.95 (3H, s, CH_3), 1.02 (3H, s, CH_3), 2.14 (m, 2H, CH_2), 3.73 (3H, s, OCH_3), 4.45 (1H, s, CH), 6.82 (2H, br s, NH_2), 6.96 (2H, br s, H-Ar), 7.14 (1H, br s, H-Ar).

FT-IR

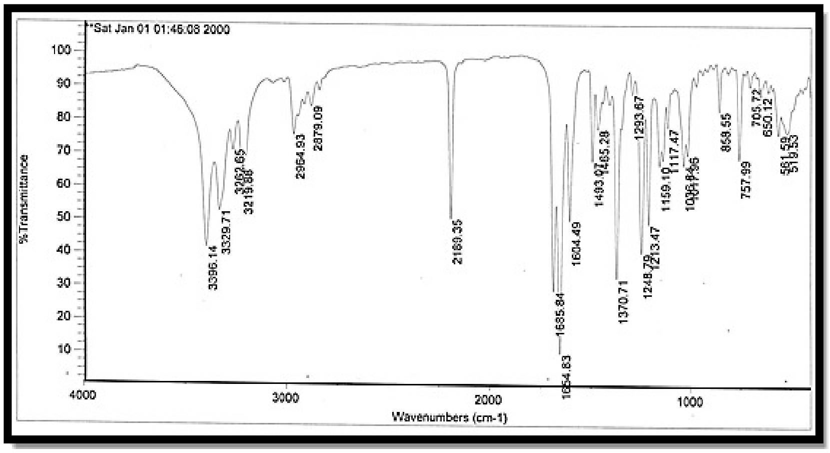

^{1}H NMR

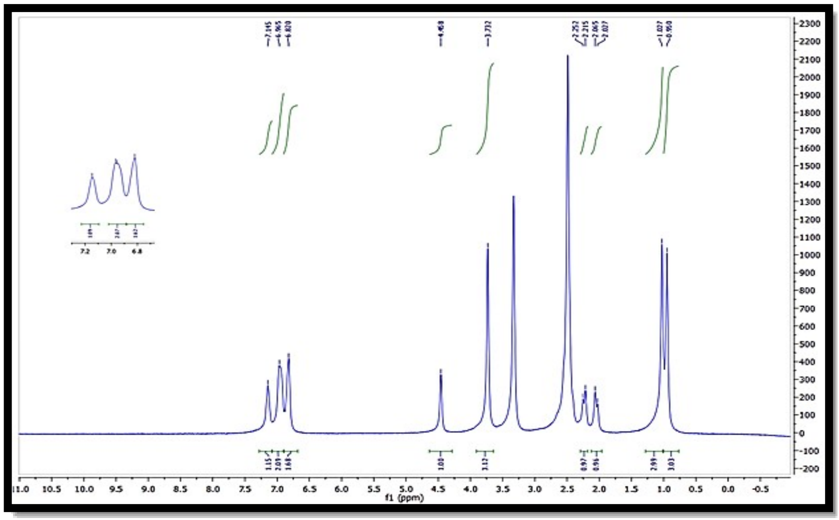

4,4'-(1,4-phenylene)bis(2-amino-7,7-dimethyl-5-oxo-5,6,7,8-tetrahydro-4*H*-chromene-3 carbonitrile)

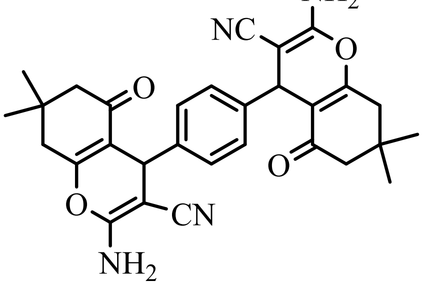

FT-IR [KBr ῡ (cm^{-1})]: 3392, 3325 (NH_2), 3254, 3211, 2963 (Ar-H), 2194 (C≡N), 1686 (C=O), 1649 (C=C), 1041 (C-O).

^{1}H NMR (400 MHz, DMSO-d_6) δppm: 0.93 (3H, s, CH_3), 1.02 (3H, s, CH_3), 2.15 (m, 2H, CH_2), 4.24 (1H, s, CH), 7.08 (2H, br s, NH_2), 7.25 (2H, d, *J*= 8, H-Ar), 7.85 (2H, d, *J*= 8, H-Ar).

FT-IR

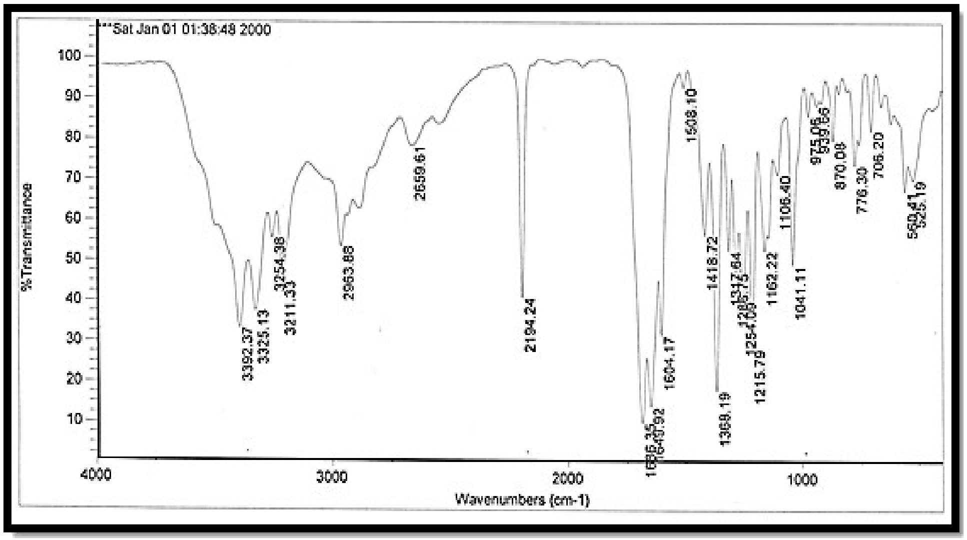

^{1}H NMR

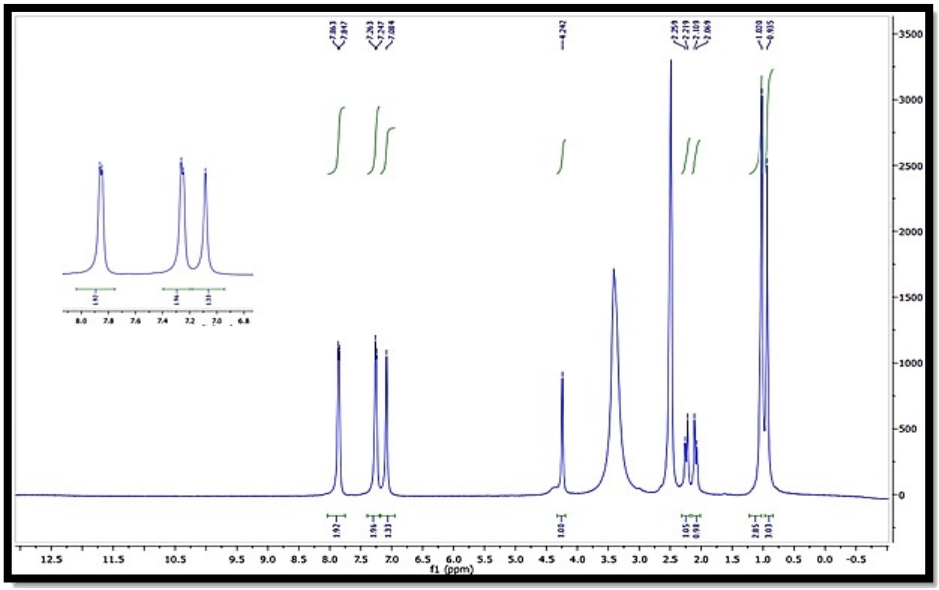

2-amino-5-oxo-4-(m-tolyl)-4,5-dihydropyrano[3,2-*c*]chromene-3-carbonitrile

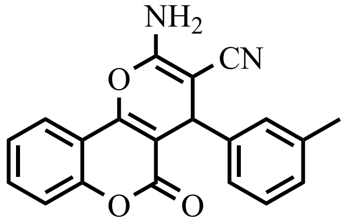

FT-IR [KBr ῡ (cm^{-1})]: 3390, 3321 (NH_2), 3050, 3015 (Ar-H), 2198 (C≡N), 1705, 1674 (C=O), 1603 (C=C), 1060 (C-O).

^{1}H NMR (400 MHz, DMSO-d_6) δppm: 2.24 (3H, s, CH_3), 4.42 (1H, s, CH), 7.03 (3H, br s, NH_2), 7.183 (1H, t, *J*= 8, H-Ar), 7.379 (1H, s, H-Ar), 7.445 (1H, d, *J* = 11, H-Ar), 7.49 (1H, d, *J* = 9.5, H-Ar), 7.69 (1H, t, *J* = 9.5, H-Ar), 7.89 (1H, d, *J* = 9.5, H-Ar).

FT-IR

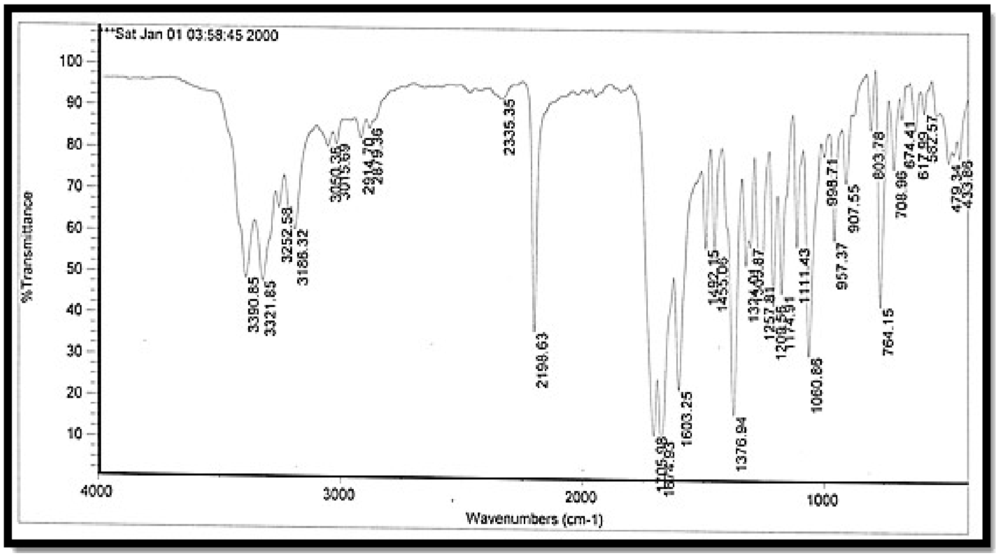

^{1}H NMR

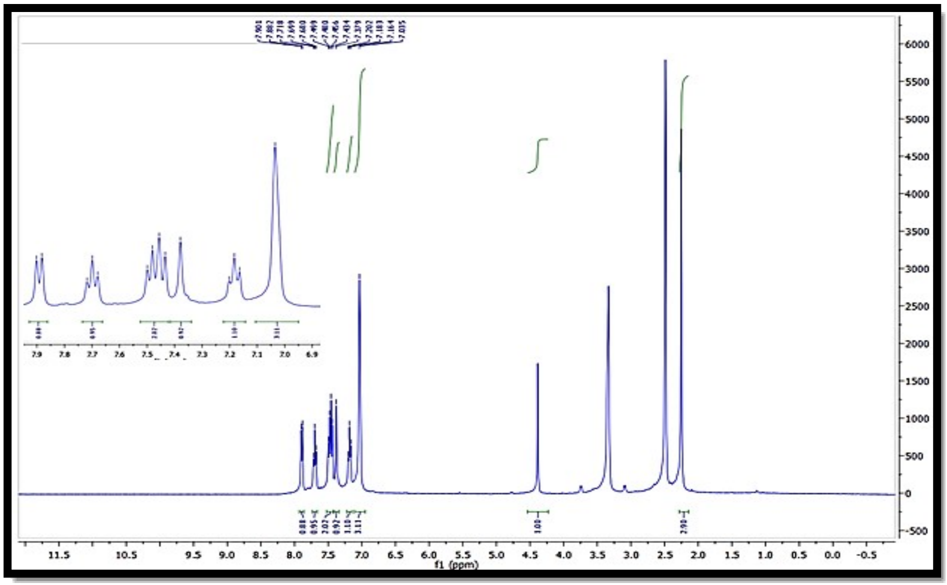

2-amino-5-oxo-4-(2-chlorophenyl)-4,5-dihydropyrano[3,2-*c*] chromene-3 carbonitrile

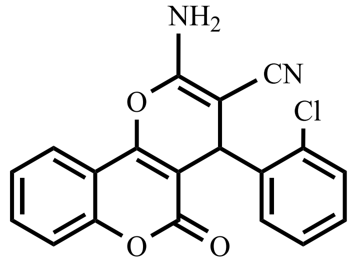

FT-IR [KBr ῡ (cm^{-1})]: 3398, 3284 (NH_2), 3179 (Ar-H), 2199 (C≡N), 1708, 1673 (C=O), 1603 (C=C), 1060 (C-O).

^{1}H NMR (400 MHz, DMSO-d_6) δppm: 4.96 (1H, s, CH), 7.28 (3H, m, NH_2, H-Ar), 7.47 (4H, m, H-Ar), 7.71 (1H, t, *J*= 9.5, H-Ar), 7.89 (1H, d, *J*= 9.5, H-Ar).

FT-IR

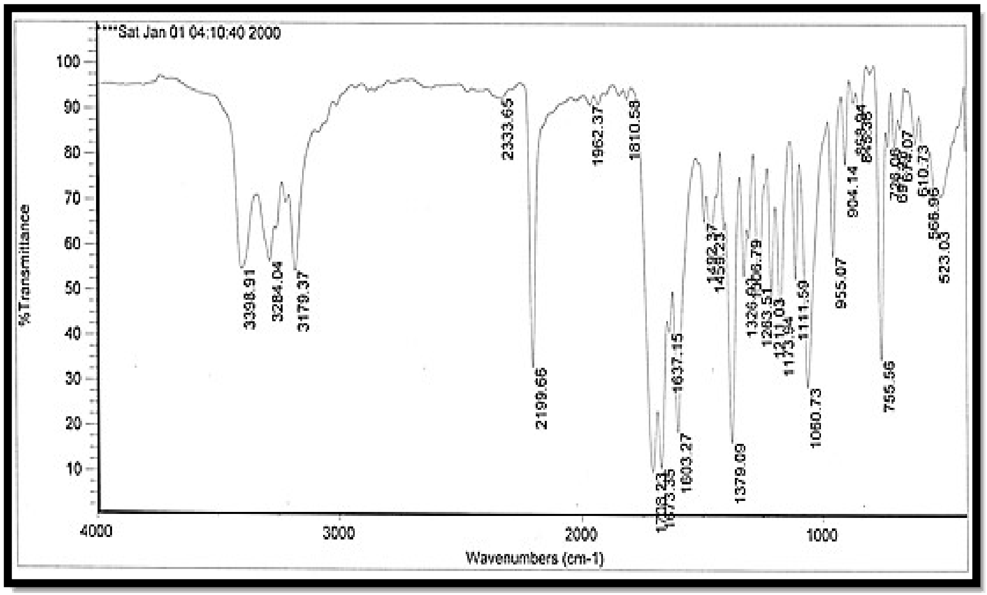

^{1}H NMR

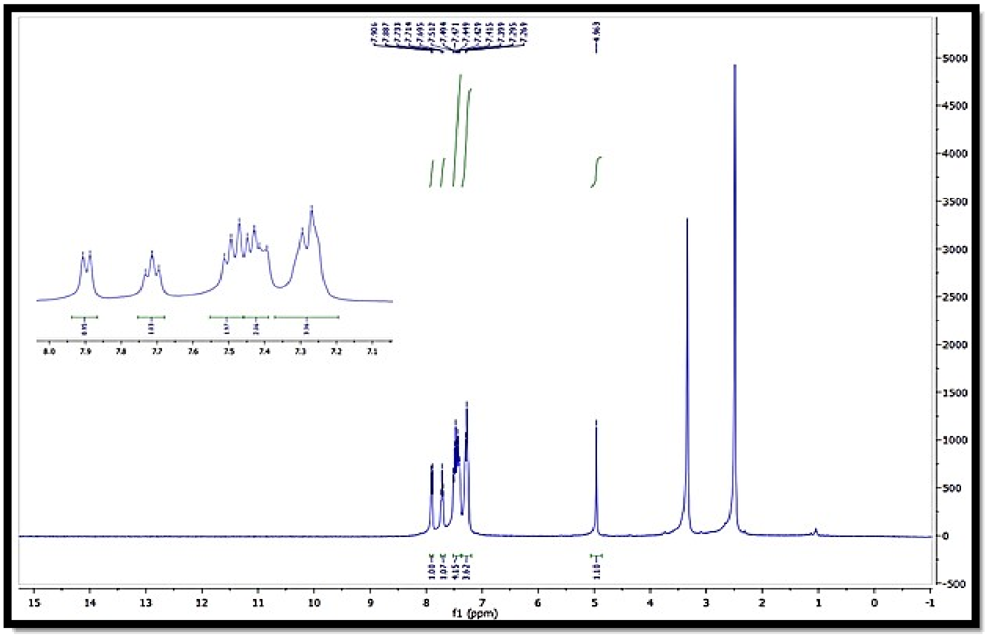

2-amino-5-oxo-4-phenyl-4,5-dihydropyrano[3,2-*c*]chromene-3-carbonitrile

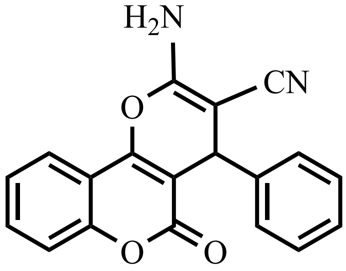

FT-IR [KBr ῡ (cm^{-1})]: 3377, 3285 (NH_2), 3180, 2887 (Ar-H), 2196 (C≡N), 1709, 1673 (C=O), 1605 (C=C), 1056 (C-O).
^{1}H NMR (400 MHz, DMSO-d_6) δppm: 4.43 (1H, s, CH), 7.24-7.47 (9H, m, NH_2, H-Ar), 7.69 (1H, br s, H-Ar), 7.88 (1H, d, *J*= 6.5, H-Ar).

FT-IR

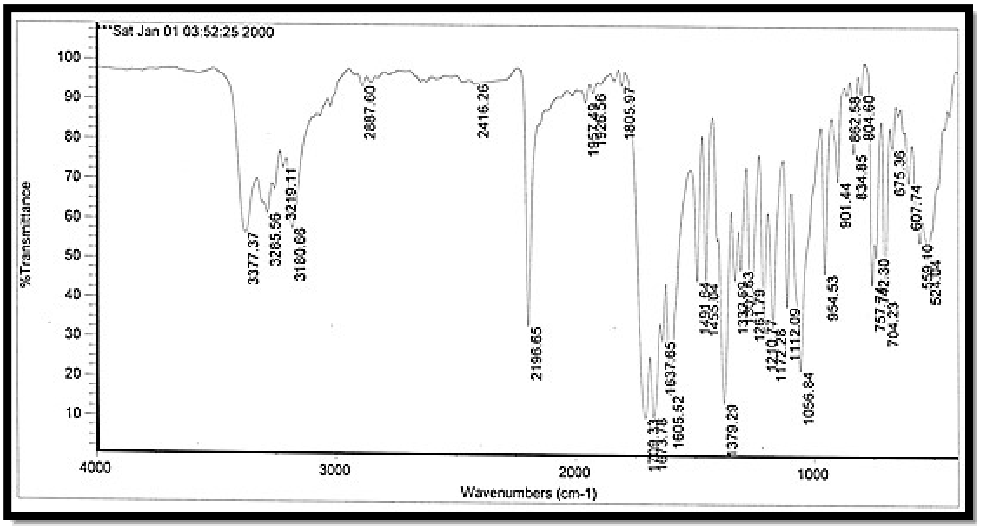

^{1}H NMR

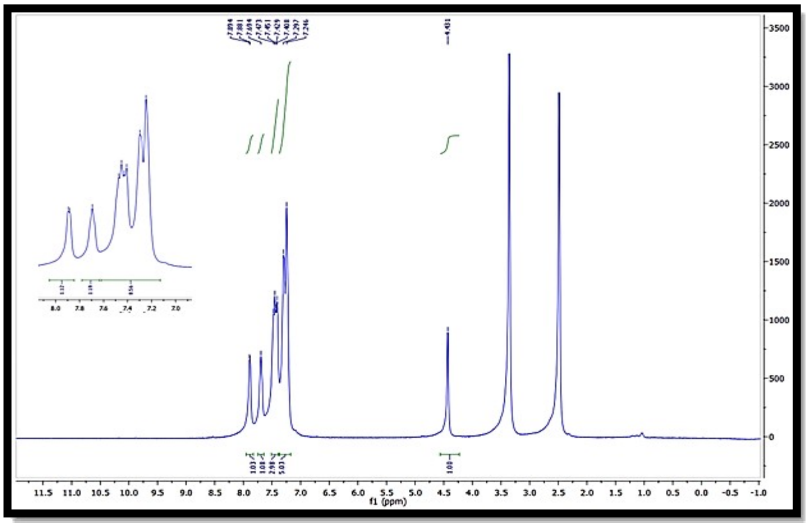

2-amino-5-oxo-4-(4-nitrophenyl)-4,5-dihydropyrano[3,2-*c*] chromene-3-carbonitrile

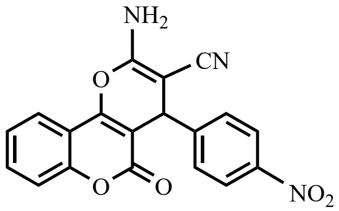

FT-IR [KBr ῡ (cm^{-1})]: 3335-3479 (NH_2), 3191, 3069 (Ar-H), 2195 (C≡N), 1718, 1672 (C=O), 1602 (C=C), 1505, 1456 (NO_2), 1054 (C-O).
^{1}H NMR (400 MHz, DMSO-d_6) δppm: 4.66 (1H, s, CH), 7.45-7.59 (4H, m, NH_2, H-Ar), 7.72 (1H, t, *J*= 10, H-Ar), 7.90 (1H, d, *J*= 10, H-Ar), 8.16 (1H, d, *J*= 10.5, H-Ar).

FT-IR

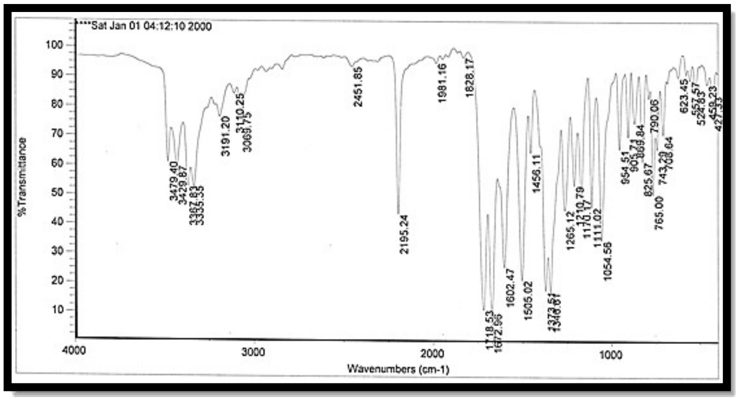

^{1}H NMR

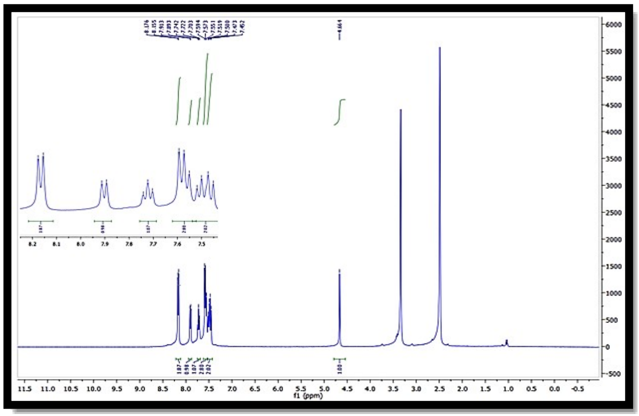

2-amino-4-(4-methoxyphenyl)-5-oxo-4,5-dihydropyrano[3,2-*c*] chromene-3-carbonitrile

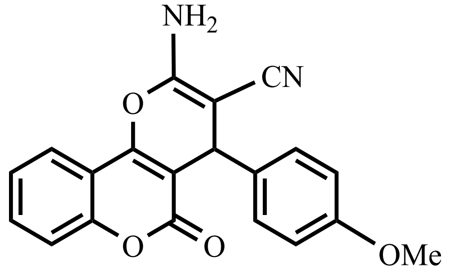

FT-IR [KBr ῡ (cm^{-1})]: 3383, 3319 (NH_2), 3252, 3190, 3064, 2954 (Ar-H), 2202 (C≡N), 1709, 1672 (C=O), 1606 (C=C), 1052 (C-O).

^{1}H NMR (400 MHz, DMSO-d$_6$) δppm: 3.70 (3H, s, OCH_3), 4.38 (1H, s, CH), 6.85 (2H, d, *J*= 11, H-Ar), 7.15 (2H, d, *J*= 11, H-Ar), 7.36 (2H, br s, NH_2), 7.43 (1H, d, *J*= 10.5, H-Ar), 7.48 (1H, d, *J*= 9.5, H-Ar), 7.69 (1H, t, *J*= 9.5, H-Ar), 7.88 (1H, d, *J*= 10, H-Ar).

FT-IR

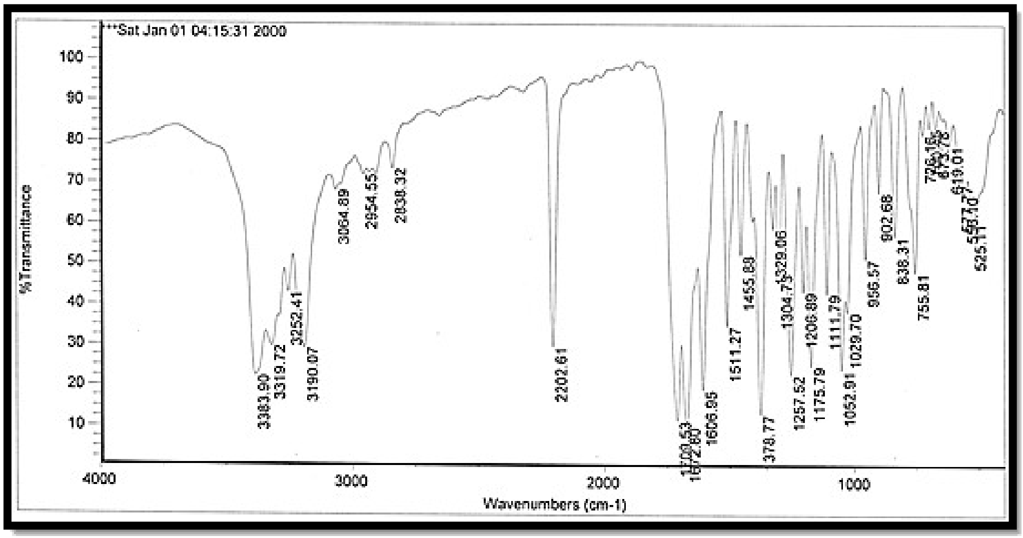

^{1}H NMR

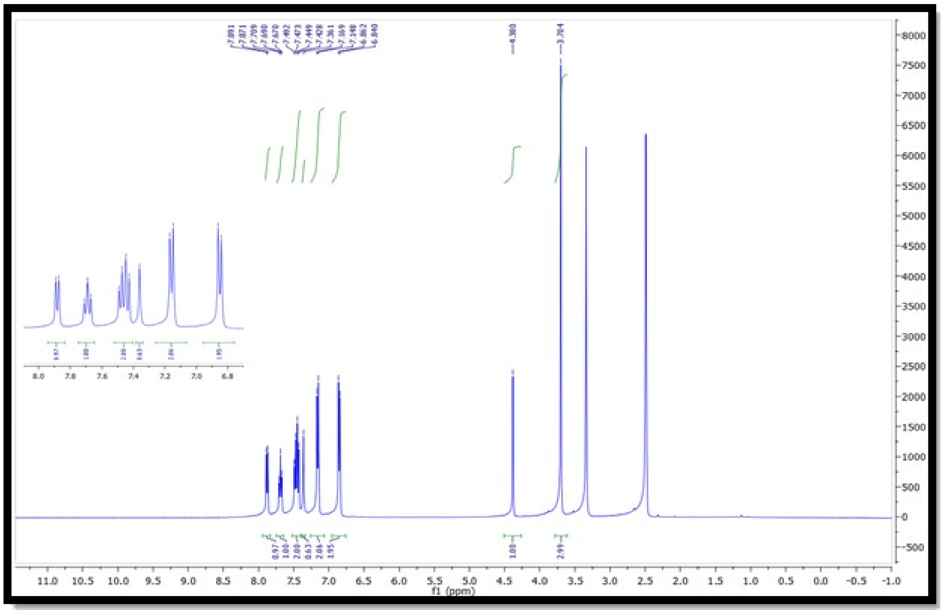

4,4'-(1,4-phenylene)bis(2-amino-5-oxo-4,5-dihydropyrano[3,2-*c*] chromene-3-carbonitrile)

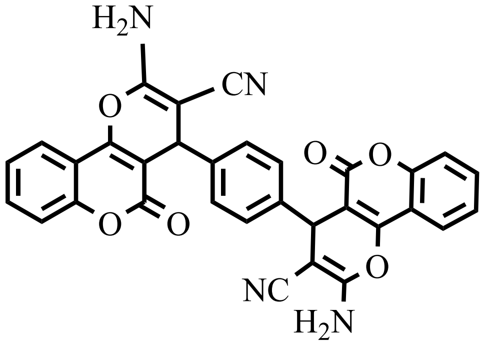

FT-IR [KBr ῡ (cm^{-1}]: 3324 (NH_2), 3191 (Ar-H), 2196 (C≡N), 1709, 1672 (C=O), 1604 (C=C), 1055 (C-O).

^{1}H NMR (400 MHz, DMSO-d_6) δppm: 4.40 (1H, s, CH), 7.26 (4H, br s, NH2), 7.31 (3H, d, J = 6.0 Hz, H-Ar), 7.42 (3H, br s, H-Ar), 7.47 (2H, d, J = 10.8 Hz, H-Ar), 7.90 (2H, d, J = 6.8 Hz, H-Ar), 7.70 (2H, br s, H-Ar).

FT-IR

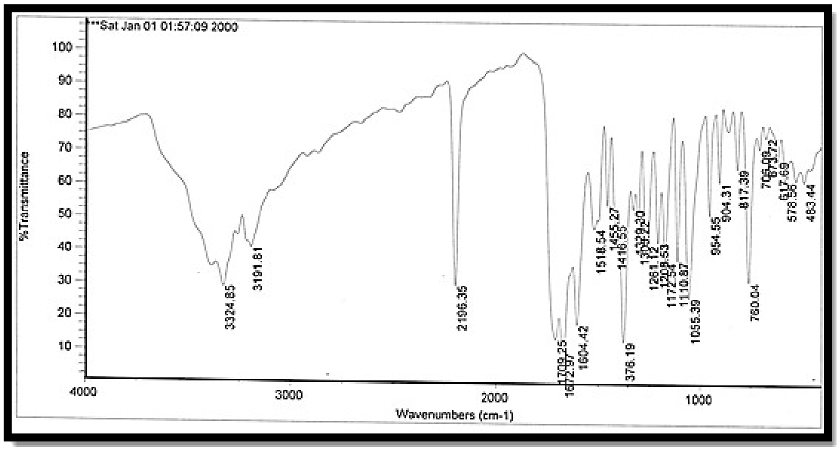
***Sat Jan 01 01:57:09 2000
%Transmittance
100
90
80
70
60
50
40
30
20
10
3324.85
3191.81
2196.35
1709.25
1672.97
1604.42
1518.54
1455.27
1416.55
1055.39
954.55
904.31
817.39
760.04
483.44
4000
3000
2000
1000
Wavenumbers (cm-1)

^{1}H NMR

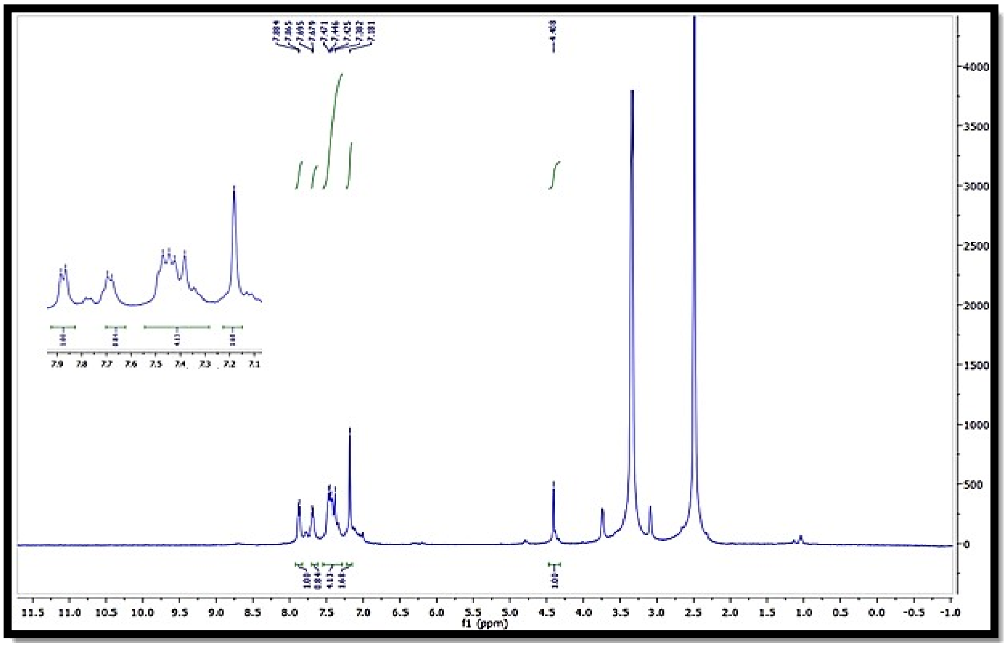
4000
3500
3000
2500
2000
1500
1000
500
0
11.5 11.0 10.5 10.0 9.5 9.0 8.5 8.0 7.5 7.0 6.5 6.0 5.5 5.0 4.5 4.0 3.5 3.0 2.5 2.0 1.5 1.0 0.5 0.0 -0.5 -1.0
f1 (ppm)
7.9 7.8 7.7 7.6 7.5 7.4 7.3 7.2 7.1

2-amino-4-(4-hydroxy-3-methoxyphenyl)-7-methyl-5-oxo-4,5 dihydropyrano[4,3-*b*]pyran-3-carbonitrile

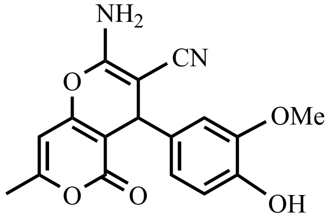

FT-IR [KBr ῡ (cm^{-1}]: 3492 (OH), 3349, 3094 (NH_2), 3094 (Ar-H), 2200 (C≡N), 1702, 1673 (C=O), 1639, 1607 (C=C), 1034 (C-O).

^{1}H NMR (400 MHz, DMSO-d$_6$) δppm: 2.22 (3H, s, CH_3), 3.74 (3H, s, OCH_3), 4.16 (1H, s, CH), 6.24 (1H, s, H-pyran), 6.51 (1H, d, J = 9, H-Ar), 6.68 (1H, d, J= 10.5, H-Ar), 6.72 (1H, s, H-Ar), 7.12 (2H, br s, NH_2), 8.92 (1H, br s, OH).

FT-IR

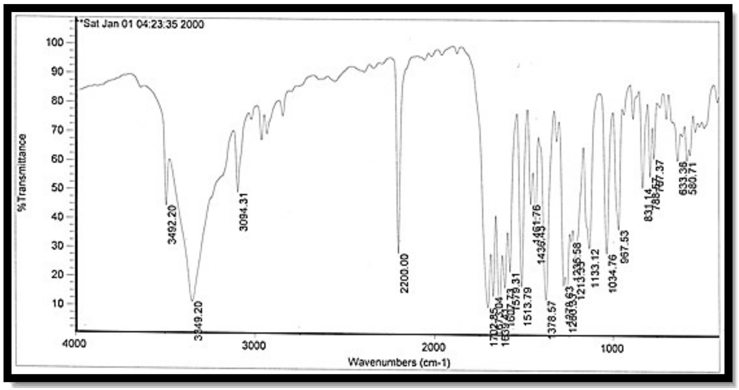

^{1}H NMR

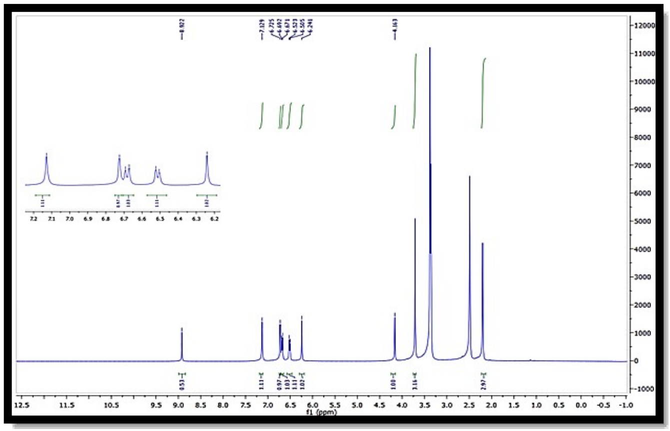

2-amino-7-methyl-4-(3-nitrophenyl)-5-oxo-4,5-dihydropyrano[4,3-b]pyran-3-carbonitrile

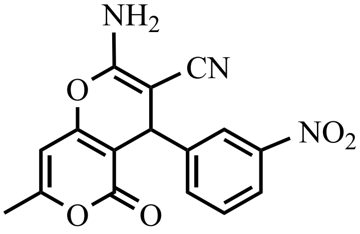

FT-IR [KBr ῡ (cm^{-1})]: 3457, 3360 (NH_2), 3237, 3184, 3118, 3076 (Ar-H), 2874, 2195 (C≡N), 1705, 1670 (C=O), 1639, 1609 (C=C), 1473, 1526 (NO_2), 1038 (C-O).

^{1}H NMR (400 MHz, DMSO-d_6) δppm: 2.21 (3H, s, CH_3), 4.54 (1H, s, CH), 6.30 (1H, s, H-pyran), 7.35 (1H, s, H-Ar), 8.03 (2H, s, NH_2), 8.11 (1H, d, *J*= 10, H-Ar).

FT-IR

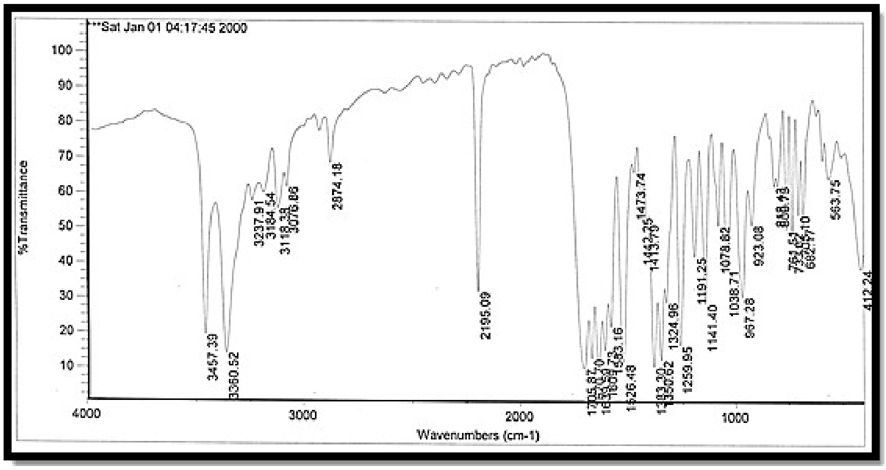

^{1}H NMR

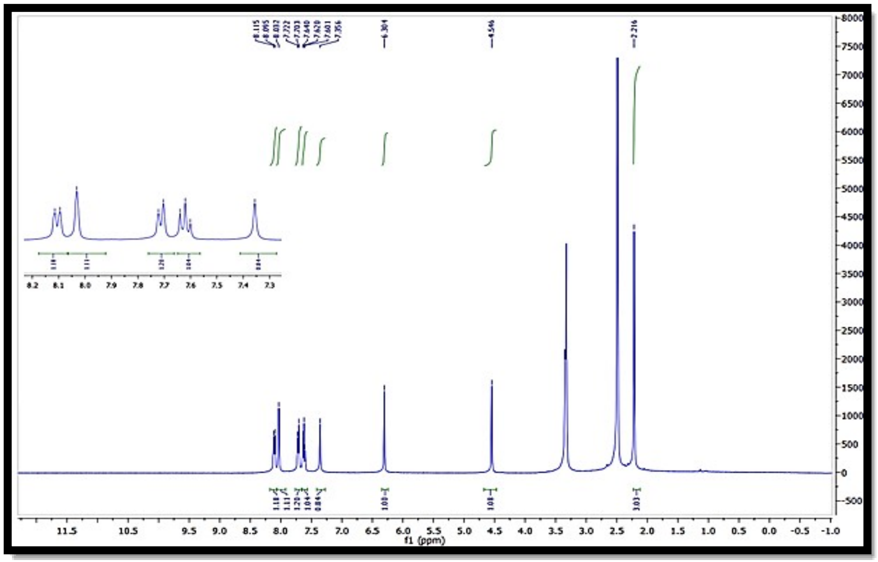

2-amino-4-(4-ethoxyphenyl)-7-methyl-5-oxo-4,5-dihydropyrano[4,3-*b*]pyran-3-carbonitrile

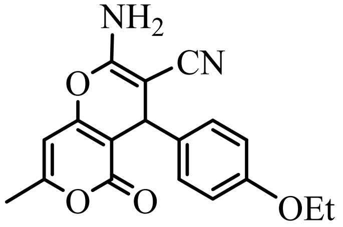

FT-IR [KBr ῡ (cm^{-1})]: 3450, 3401 (NH_2), 3209 (C-H), 3101, 2980 (Ar-H), 2929, 2883, 2195 (C≡N), 1702, 1671 (C=O), 1643, 1610 (C=C), 1042 (C-O).
^{1}H NMR (400 MHz, DMSO-d_6) δppm: 1.28 (3H, t, *J*= 8, CH_3), 2.19 (3H, s, CH_3), 3.96 (2H, q, *J* = 7.5, CH_2), 4.19 (1H, s, CH), 6.24 (1H, s, H-pyran), 6.82 (2H, d, *J*= 10, H-Ar), 7.06 (2H, d, *J* = 10, H-Ar), 7.14 (2H, br s, NH_2).

FT-IR

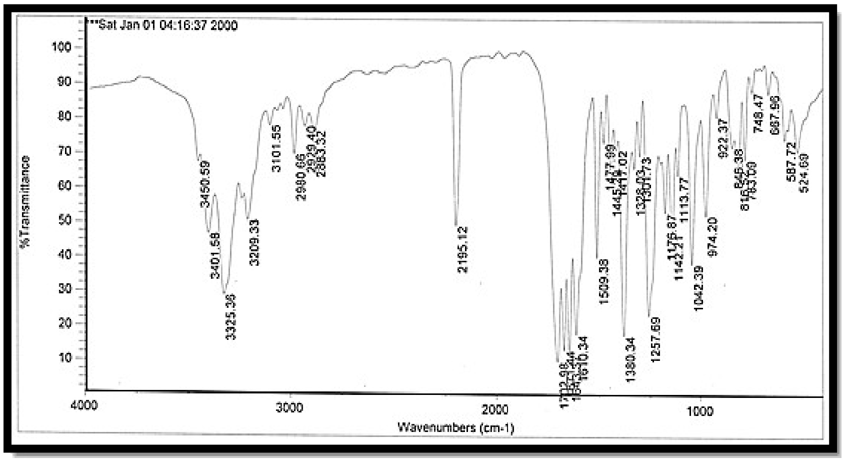

^{1}H NMR

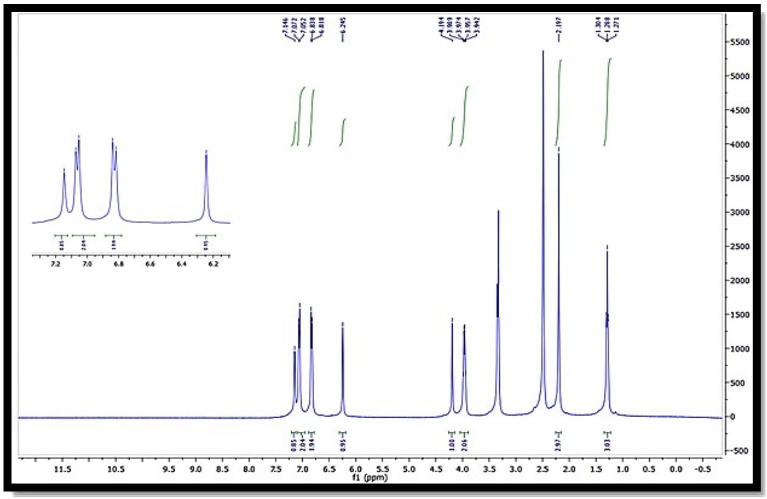

4,4'-(1,4-phenylene)bis(2-amino-7-methyl-5-oxo-4,5-dihydropyrano [4,3-b]pyran-3-carbonitrile)

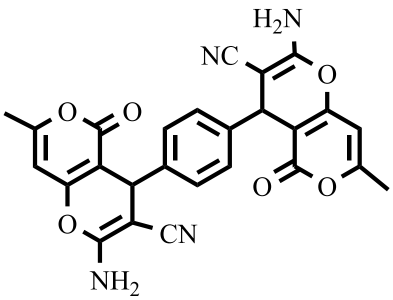

FT-IR [KBr ῡ (cm^{-1})]: 3457, 3323 (NH_2), 3196 (Ar-H), 2891 (aliphatic C-H), 2195 (C≡N), 1704, 1677 (C=O), 1643, 1610 (C=C), 1039 (C-O).
^{1}H NMR (400 MHz, DMSO-d_6) δppm: 2.19 (3H, s, CH_3), 4.22 (1H, s, CH), 6.25 (1H, s, H-pyran), 7.09 (2H, s, NH_2), 7.18 (4H, s, H-Ar).

FT-IR

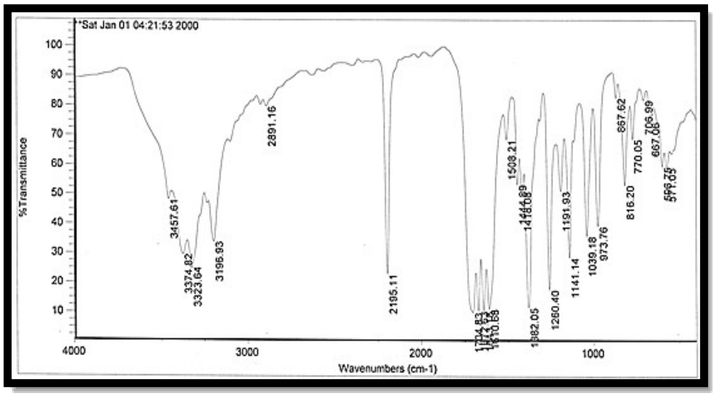

^{1}H NMR

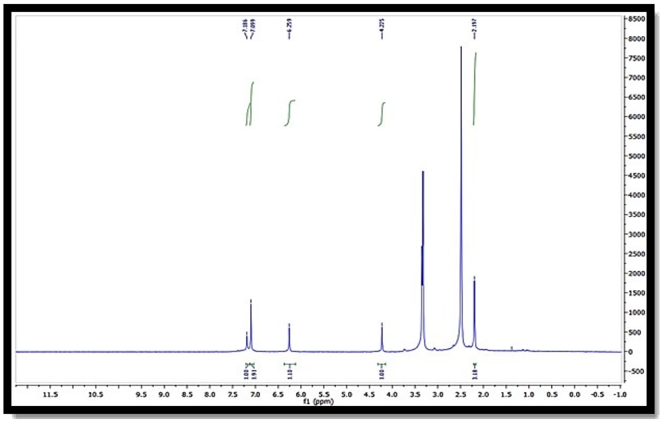

3-amino-1-(3-nitrophenyl)-1H-benzo[f]chromene-2-carbonitrile

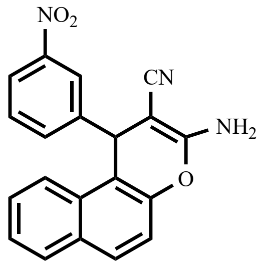

FT-IR [KBr ῡ (cm^{-1})]: 3404, 3356 (NH_2), 3196, 2891 (Ar-H), 2190 (C≡N), 1658 (C=O), 1643, 1610 (C=C), 1527, 1349 (NO_2), 1083 (C-O).

^{1}H NMR (400 MHz, DMSO-d_6) δppm: 5.40 (1H, s, CH), 7.20 (2H, s, NH_2), 7.42 (2H, d, *J* = 9.01 Hz, Ar-H), 7.51–7.57(2H, m, Ar-H), 7.63 (1H, d, *J* = 7.74 Hz, Ar-H), 7.78 (1H, d, *J* = 9.11 Hz, Ar-H), 7.93 (1H, d, *J* = 9.06 Hz, Ar-H), 8.01 (1H, d, *J* = 8.01 Hz, Ar-H), 8.06 (1H, s, Ar-H), 8.05 (1H, d, *J* = 1.75 Hz, Ar-H).

FT-IR

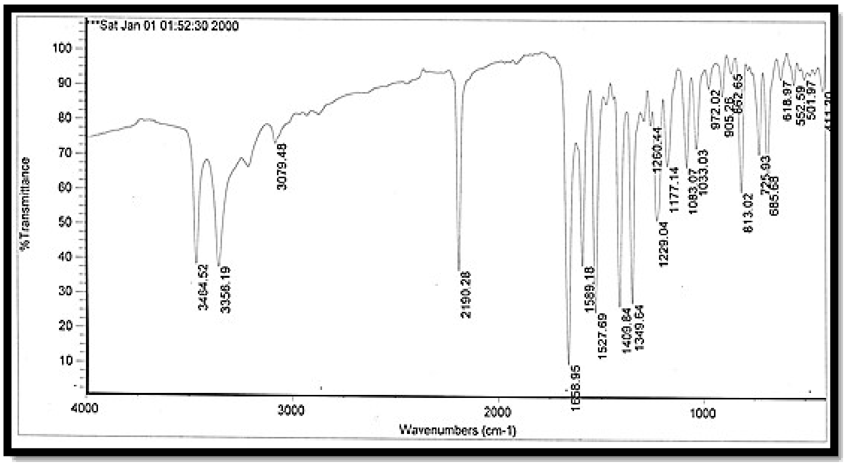

^{1}H NMR

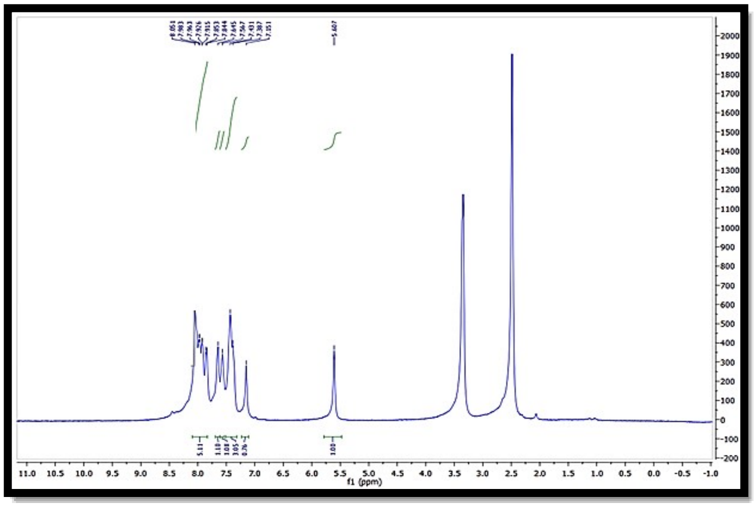

3-amino-1-(p-tolyl)-1*H*-benzo[*f*]chromene-2-carbonitrile

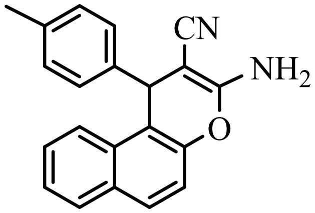

FT-IR [KBr ῡ (cm^{-1})]: 3404, 3356 (NH_2), 3079 (Ar-H), 2190 (C≡N), 1658, 1615 (C=C), 1035 (C-O).

^{1}H NMR (400 MHz, DMSO-d$_6$) δppm: 2.17 (3H, s, CH_3), 5.22 (1H, s, CH), 6.25 (1H, s, CH), 6.94 (1H, br s, H-Ar), 7.04 (4H, br s, H-Ar), 7.30-7.40 (8H, m, H-Ar, NH_2), 7.80-7.89 (3H, m, H-Ar).

FT-IR

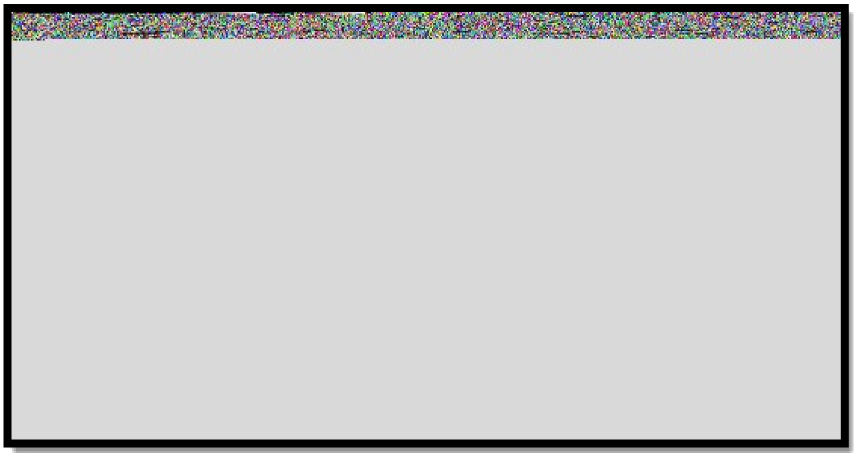

^{1}H NMR

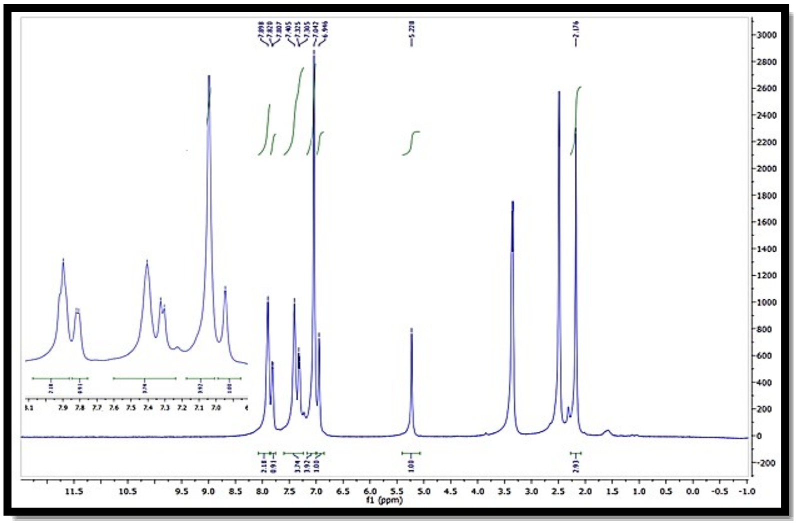

3-amino-1-(2,4-dichlorophenyl)-1*H*-benzo[*f*]chromene-2-carbonitrile

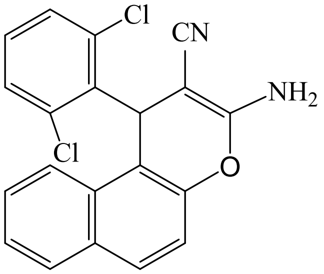

FT-IR [KBr ῡ (cm^{-1})]: 34093, 3356 (NH_2), 2156 (C≡N), 1684, 1450 (C=C), 1254(C-O).

^{1}H NMR (400 MHz, DMSO-d_6) δppm: 5.20 (1H, s, CH), 7.00-8.00 (11H, m, H-Ar, NH_2).

FT-IR

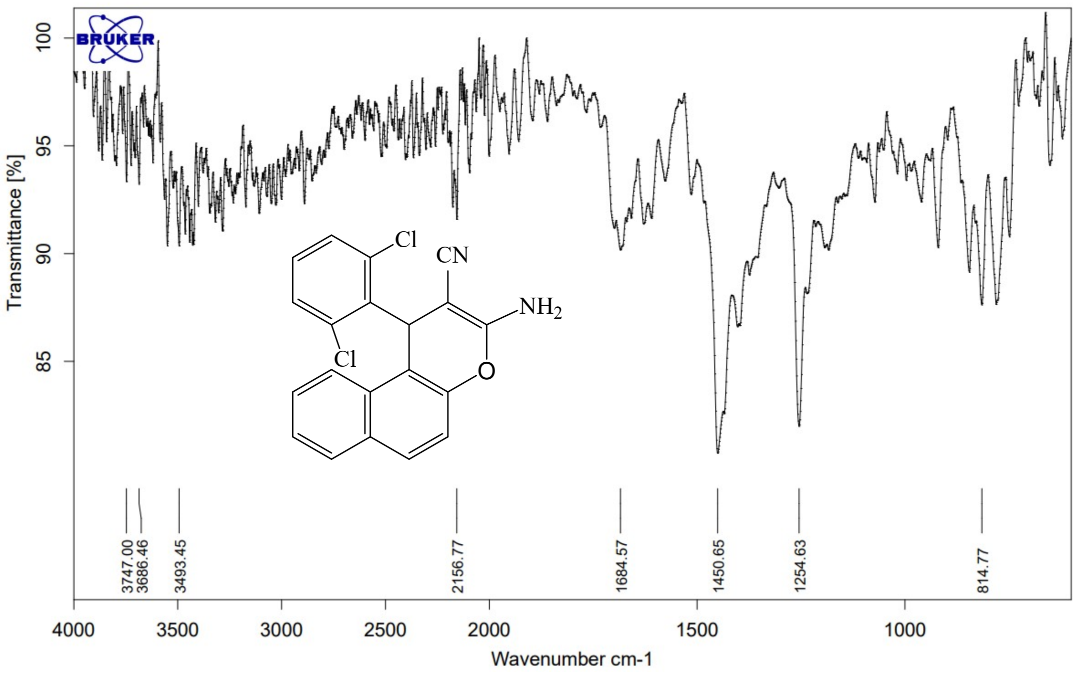

^{1}H NMR

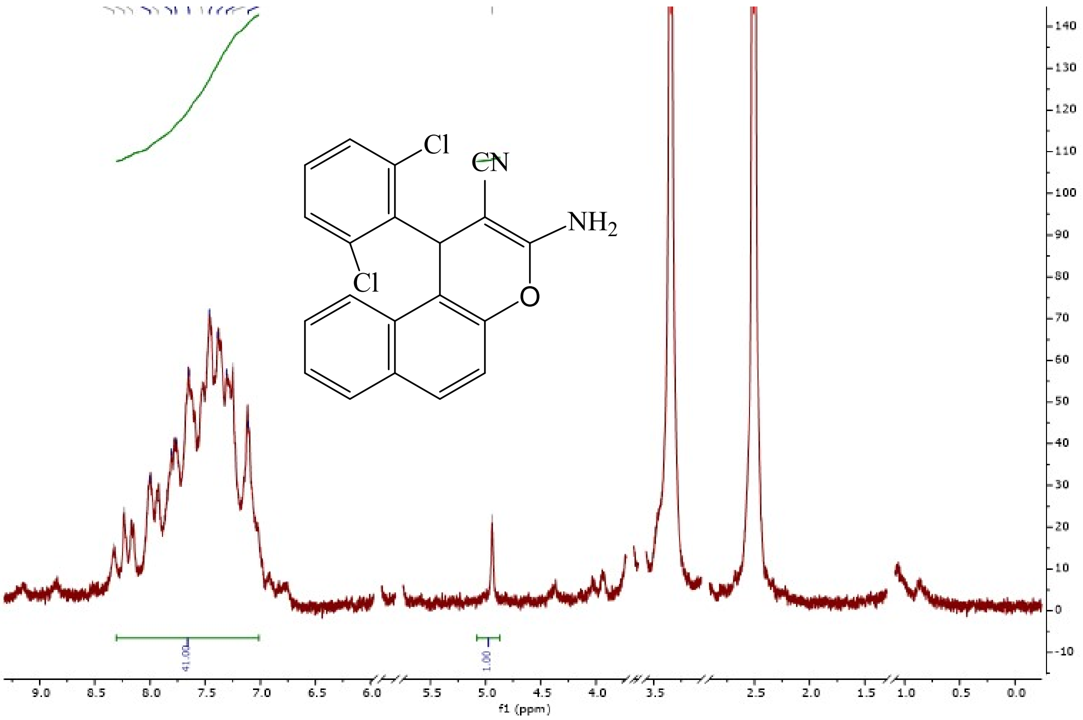

Supplement: RA-012-D2RA05057A-s001 [file RA-012-D2RA05057A-s001.pdf]
